# Experimental exploration of fracture behavior (pure mode III) in eco-friendly steel fiber-reinforced self-compacting concrete with waste tempered glass as coarse aggregates

**DOI:** 10.1038/s41598-024-58912-z

**Published:** 2024-04-19

**Authors:** Pooyan Pournoori, Amirhossein Davarpanah T.Q., Arash Rajaee, Morteza Ghodratnama, Saeed Abrishami, Amir R. Masoodi

**Affiliations:** 1 Department of Civil Engineering, Khayyam University of Mashhad, Mashhad, Iran; 2 Department of Civil Engineering, Eqbal Lahoori Institute of Higher Education, Mashhad, Iran; 3 Department of Civil Engineering, Ferdowsi University of Mashhad, Mashhad, Iran

**Keywords:** Eco-friendly self-compacting concrete, Waste glass coarse aggregate, Steel fibers, Fracture toughness parameters, ENDB specimen, Engineering, Civil engineering

## Abstract

To aid in the creation of sustainable structures, scientists have utilized waste materials found in the environment to serve as alternatives for traditional resources in the construction sector. They have undertaken extensive investigations pertaining to this matter. In this particular study, tempered glass as waste glass coarse aggregate (WGCA) was substituted for natural coarse aggregate (NCA) at varying proportions of 15%, 30%, and 45% in the formulation of eco-friendly self-compacting concrete (SCC), combined with hooked-end steel fibers (SFs) at various volumes. The study assessed concrete’s flowability, permeability, compressive strength, and fracture parameters at 28 and 56 days. A total of 240 edge-notched disc bending samples (ENDB) and 60 cubic samples (150 × 150 mm) were tested to assess fracture resilience and compressive strength, respectively. The results showed that increasing SF and WGCA content reduced slump flow diameter and blockage ratio, particularly at higher levels. The solidified characteristics of all specimens incorporating SF and WGCA displayed heightened attributes when contrasted with the reference sample. Among the entire array of specimens, WG15SF0.5 and WG30SF0.5 exhibited the most superior performance, demonstrating an average percentage elevation of 20.29 and 27.63 in both compressive strength and fracture toughness assessments across the different curing periods. SF had the most significant impact on post-cracking behavior by enhancing load-bearing capacity through a bridging fiber mechanism. Through a comparison of the influence of SFs and WGCA on the fracture toughness of pure mode III, it was observed that the inclusion of SF in samples with a 30% replacement of WGCA resulted in an average increase of approximately 15.48% and 11.1% in this mode at the ages of 28 and 56 days, respectively, compared to the control sample.

## Introduction

Concrete is a commonly utilized material in the construction industry due to its availability, affordability, substantial compressive strength, formability when molded, and the ability to modify its strength through changes in the proportion of its components (such as cement, water, and aggregate). Depending on the nature of the project, different types of concrete may be used. For instance, with the advancement of technology and the design of complex concrete structures, there is a growing demand for concrete possessing high workability that can easily consolidate congested reinforced concrete members^1^. Consequently, apart from addressing these problems, the concrete being considered must have sufficient durability and strength, leading to the development of high-performance concretes (HPCs). As part of this effort, a specific type of HPC known as self-compacting concrete (SCC) was created^2^. H. Okamura proposed the SCC back in 1986^3^. One of the primary benefits of SCC is that it can self-consolidate without experiencing the separation of constituents, only under the effect of its own weight^4^. This happened because more cement content is used in SCC compounds than in other types of concrete, such as normal concrete and RCD (Roller Compacted concrete for Dams)^3^. In this regard, the researchers proposed adding supplementary cementitious materials (SCM) such as fly ash, metakaolin, silica fume, blast furnace slag, and limestone powder to replace some of the cement in the mixture^5^. This practice enhances the rheological properties of SCC, diminishes production costs, and lowers the temperature of the hydration process. Similarly, numerous investigations conducted by researchers have explored various characteristics of self-compacting concrete (SCC), such as its mechanical properties^6,7^, durability^8,9^, permeability^10,11^, heat resistance^12,13^, and fracture mechanics parameters^14–16^.

Previous studies have shown that one of the concrete’s main weaknesses is its low capacity to resist tension and crack growth (fracture toughness)^17^. As a result, this concrete nature can lead to brittle fracture when loaded. However, in the field of civil engineering, most designs aim for ductile fracture in structures. Concrete elements that exhibit this property have improved attributes, such as higher ductility and strength toughness, increased durability, better matrix stiffness, and increased hardening in the post-crack response compared to the brittle state^18,19^. Researchers have explored the use of various fibers in concrete to address this issue. Among these fibers, steel fibers have gained significant attention and investigation due to their strength, high elastic modulus, toughness, and reasonable price in the construction of different types of concrete^1,20^. Furthermore, in projects where controlling crack growth or enhancing concrete’s cracking resistance is a priority, steel fibers are used instead of synthetic fibers^21^. The placement of steel fibers near a crack surface helps to increase the residual tensile strength capacity of that area by acting as a fiber bridging mechanism^22^. This is because steel fibers demonstrate strong resistance to cutting in wide cracks. Boulekbache et al. found in their research that steel fibers enhance the ductility of both normal and self-compacting concrete more significantly than high-strength concrete (HSC)^23^. This is because these two concretes have better flowability than HSC. Pajak and Ponikiewski stated that the increase in fracture energy of SFR-SCC samples containing hooked-end steel fibers was greater than the specimens including straight steel fibers^24^. Nehme et al. claimed that incorporating hooked-end steel fibers in the steel-mesh-reinforced SCC panel significantly enhanced its load–deflection capacity, load-deformation values, and ultimate load capacity^25^.

In recent decades, the surge in concrete consumption within the construction sector and the constrictions imposed by finite natural resources have compelled researchers to explore alternative materials for replacing cement and aggregates in concrete and mortar blends. This pursuit aims to avert environmental degradation and conserve the integrity of the environment and building materials. Consequently, numerous investigations have delved into the substitution of conventional coarse and fine natural aggregates with recycled aggregates, such as recycled concrete aggregates^26,27^ and recycled plastics^28,29^, as well as waste aggregates, including glass waste aggregates^30–32^ and ceramics^33,34^, across various concrete types and mortar compositions. Amid these efforts, the expense associated with glass recycling, the imperative to curb the accumulation of glass industry waste, and the challenge of its non-biodegradability have driven the exploration of employing waste glass in diverse applications. This encompasses the incorporation of waste glass in the production of concrete, mortar, building blocks, sub-bases, and asphalt^31,35,36^. Gautam et al. documented that the replacement of up to 40% of fine-grain waste glass in architectural concrete does not lead to a significant reduction in concrete strength^37^. In comparison to natural aggregates, glass exhibits notably minimal water absorption. Consequently, waste glass coarse aggregate remains uninvolved in the alkali-silica reaction (ASR) that occurs between aggregates and cement^38,39^. This characteristic also contributes to a reduction in concrete shrinkage^40^. The incomplete adhesion of waste glass coarse aggregate (WGCA) to the cement binder results in diminished concrete strength and compromises other mechanical properties^41^. Polly et al. attributed this effect to the inherently poor shape, poor surface characteristics, and high brittleness of WGCA^42^. Kou and Poon's research revolved around the utilization of waste glass aggregate (WGA) as a replacement for natural fine aggregate (at 10%, 20%, and 30%) and coarse aggregate (at 5%, 10%, and 15%) within self-compacting concrete mixtures. Their findings demonstrated that an increase in WGA content led to fresh properties of SCC, such as enhanced slump flow, reduced blockage ratio, and increased air content. However, the impact of elevated WGA content on the hardened properties of SCC resulted in a reduction in static modulus of elasticity, compressive strength, and tensile splitting strength^43^. Mousavi et al. reported that the optimal replacement percentage for waste glass fine aggregate in self-compacting mortar, resulting in a 20–40% increase in sample fracture toughness, was highlighted^44^. Kuri et al. conducted a study involving the incorporation of waste glass coarse aggregate (WGCA) with a maximum aggregate size of 19 mm, replacing up to 40% of the natural coarse aggregate in ordinary Portland cement concrete (OPC). Their findings revealed that while the augmentation of WGCA led to a reduction in slump, compressive strength, and drying shrinkage, it also yielded an escalation in chloride permeability and porosity^45^. They attributed this phenomenon to the coarse-grained nature of glass, characterized by a smooth and impermeable surface. This characteristic prompts the accumulation of free water on the glass surface within the concrete mixture, thereby hindering the adhesion and continuous integration between waste glass aggregate (WGA) and the cement matrix. Subsequently, as processing takes place, the water in the interfacial transition zone (ITZ) evaporates, giving rise to the formation of pores. They deemed it preferable to substitute WGCA in concrete up to a maximum of 20%.

Cracking in concrete structures generally leads to negative outcomes, including the transfer of moisture and harmful ions, damage to the cement paste, and an increase in the carbonation of the hydration products. These factors ultimately lead to a reduction in the sustainability and durability of these structures. However, studying the behavior of concrete cracks and understanding their characteristics is a complex task that requires accurate and proper evaluations^46^. In materials, cracks typically occur in three principal deformation modes, namely mode one (Opening or Tension), mode two (Sliding or In-plane shear), and mode three (Tearing or Anti-plane shear)^44,46,47^. In Fact, cracking occurs in engineering components under the effect of the mixed modes load^47^, However, fracture can occur in pure modes or as a result of mode interactions. The initiation and propagation of cracks are more commonly influenced by combined modes such as I–II and I–III^48–50^. While the fracture toughness of mode III is crucial for engineers, as different structural elements are subject to torsional loading, obtaining the material's resistance factor against crack growth in mode III (K_IIIc_) is essential^51^. To this end, researchers have conducted both theoretical and experimental studies to fully comprehend the fracture mechanics parameters of various materials, including fracture toughness, as noted in the literature^52–55^.

Despite several methods available for determining fracture parameters, a lack of uncomplicated and straightforward test procedure for determining the critical mode III stress intensity factor (K_IIIc_) for different concrete samples persists. To address this issue, Aliha et al. introduced a new model called Edge Notched Disc Bendِ (ENDB), which can test and calculate the fracture toughness of composite materials like concrete and asphalt in pure modes III, I, and the combination of these modes (I/III or III/I)^56,57^. The authors noted that mode I fracture toughness of ENDB samples was consistent with similar samples from other tests. Additionally, they discovered that K_IIIc_ values for all samples were smaller than K_Ic_ values^56^. In another study, ENDB samples were utilized to explore the fracture toughness of concrete composites that contained synthetic fibers, and it was found that the impact of fibers on mode III fracture toughness was significantly greater than that of mode I^58^. Additionally, the researchers observed that the K_Ic_ value was higher than the corresponding K_IIIc_ value for all samples, but the fracture energy (G_f_) value for pure mode III samples was significantly greater than that of pure mode I samples. Kazemian et al. conducted a study on substituting natural coarse with recycled concrete aggregates in both Treated and Untreated forms (at levels of 25% and 50%) in concrete compositions. They found that the K_Ic_ and K_IIIc_ values obtained from the ENDB sample decreased as the replacement ratio of aggregates increased, with a more pronounced decrease in the K_Ic_ value^59^. Hoseini et al. investigated the impact of increasing the volumes of coarse aggregates (at levels of 30%, 40%, 50%, and 60%) and steel fibers (at levels of 0.15%, 0.3%, and 0.45%) on fracture modes I and III of self-compacting concrete using ENDB samples. They discovered that increasing amounts of coarse aggregates up to 50% increased the fracture toughness in both pure modes. However, increasing this value up to 60% decreased the fracture parameters of the concrete samples^60^. It is worth noting that among the main components of concrete (cement, water, and aggregate), the aggregate plays a crucial role when the concrete is cracked. This is because it contributes to the initiation of cracks, stress concentration, and the limitation of crack growth.

As previously stated, predicting the performance of different types of concrete and mortar under varying conditions is a complex and challenging task. Therefore, the investigation and exploration of this field require a comprehensive understanding of engineering science. To this end, a comprehensive study was conducted to assess the primary influence of fibers, especially steel fibers, on concrete to examine how fibers can regulate crack propagation and enhance the capacity of concrete to withstand cracking. The study also highlighted the need to use waste glass as an alternative to natural aggregates and to evaluate its impact on the properties of concrete and mortar. The main aim of this research is to employ waste glass accumulation as a replacement for natural aggregates (NCA) in concrete, while also incorporating steel fibers to enhance fracture behavior, particularly in the context of torsional mode, within concrete containing WGCA. The researchers noted that previous studies had not examined the combined effect of steel fibers and waste tempered glass coarse aggregate on the final stress intensity factor of modes I and III of self-compacting concrete. Therefore, the present study aimed to investigate the impact of adding Hooked end steel fibers at Volume fraction of 0.1%, 0.3%, and 0.5%, as well as waste glass coarse aggregate at replacement percentages of 15%, 30%, and 45%, instead of natural coarse aggregate, on the fresh properties (Slump flow, L-Box), compressive strength, and self-compacting concrete failure parameters, including pure modes I, III, and their combination, using ENDB samples at processing ages of 28 and 56 days. The outcomes verify the viability to substitute WGCA by up to 45% in place of NCA within SFR-SCC specimens. These findings demonstrated a decline in the fluidity and passage capability of the mixture, coupled with a rise in compressive strength. Furthermore, a noteworthy enhancement in concrete's fracture toughness parameters was observed at both 28 and 56 days, particularly when supplemented with fibers at volume fractions ranging from 0.1 to 0.5%. The results also indicated that variations in specimen age and the nature of loading modes influenced the patterns of crack growth and propagation, as well as the surface characteristics.

## Experimental details

### Materials

#### Cement and limestone powder

Type 1 Portland cement, having a specific density of 3150 (kg/m^3^) and a specific surface area of 3000 (cm^2^/gr) in accordance with ASTM C150^61^, was employed. In this research, an additive called limestone powder (LSP) with a specific surface area of 2620 (cm^2^/gr), as per ASTM C188-89^62^, was incorporated. The purpose of using LSP was to enhance the fluidity, cohesion, resistance to segregation, and the proportion of fine powder content in the mixing design.

#### Natural aggregates

**Figure 1:**
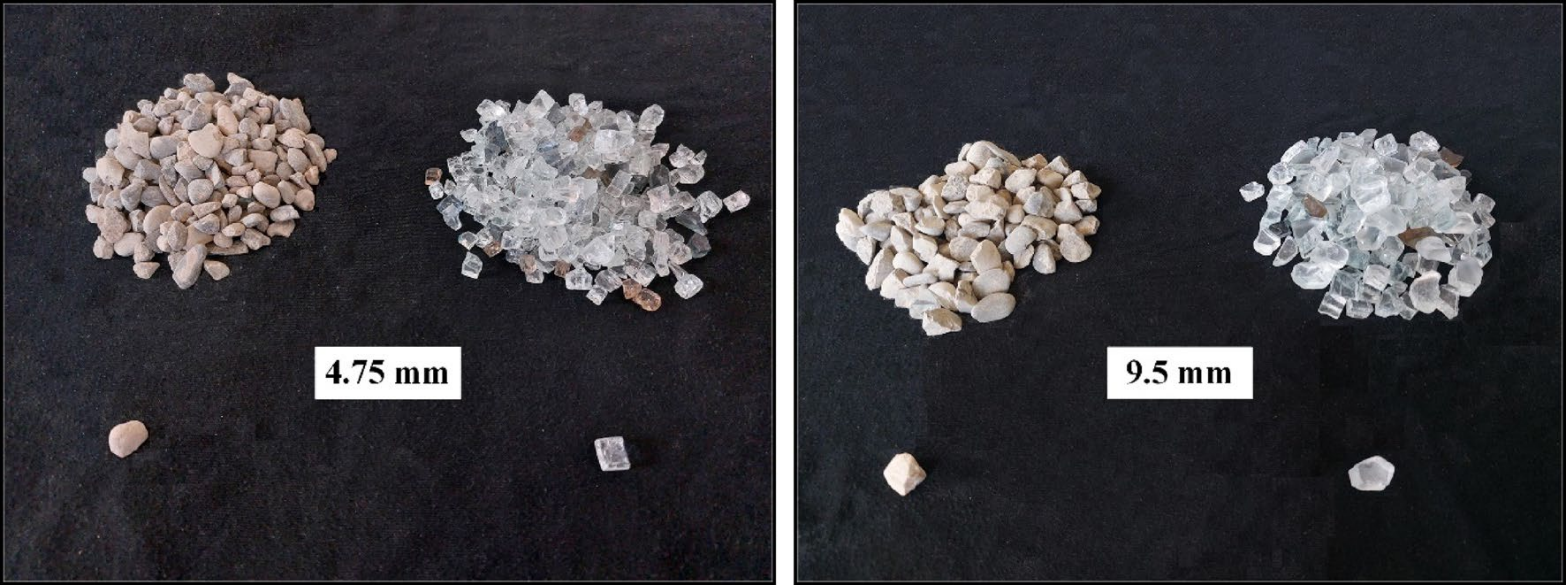
Coarse aggregates of NCA and WGCA used for casting SFR-SCC mixtures.

River-type natural aggregates were utilized in all the mixtures. The maximum size of the natural coarse aggregate was 9.5 mm. The water absorption percentages for the natural coarse and fine aggregates were 0.87 and 1.42, respectively. The fine aggregate had a modulus of elasticity of 2.67. Table 1 presents the physical properties of the natural aggregates, including their granulation distribution, as per ASTM C33^63^. Figure 1 illustrates the visual appearance of the natural coarse aggregates (NCA).

**Table 1:** Particle size distribution and physical characteristics of consuming aggregates.

Sieve size (mm)	Natural fine aggregate (% passing)	Coarse aggregate	
		Natural (% passing)	Waste glass (% passing)
12.5	–	100	100
9.5	–	49	49
4.75	100	0	0
2.36	95	–	–
1.18	77	–	–
0.6	42	–	–
0.3	19	–	–
0.15	0	–	–
Relative density (gr/cm^3^)	2.65	2.76	2.46
Water absorption (%)	1.42	0.87	0.09
Angularity	Semi angular or rounded	Semi angular or rounded	Very angular

#### Waste tempered glass

The waste glass utilized in this research is tempered glass, sourced from the waste repository of Shishe Imeni Shargh Mashhad Safety Glass Factory. Tempered glass, after fracturing, can be categorized into two types: the initial type is transformed into small grains that can be directly employed after undergoing washing and drying. The second type is fragmented into plate-like shapes but remains partially intact^64–66^. Siddique et al. research exploring the influence of substituting waste tempered glass fine aggregates (ranging from 5 to 20%) in ordinary concrete demonstrated an overall increase in compressive strength across all mixtures containing waste glass, as compared to the reference sample^67^. In this current study, the first variant of tempered glass was utilized. After the collection of the glass, it underwent washing and drying procedures. Subsequently, the glass was sifted based on the intended granulation. Ultimately, the prepared waste glass aggregates were incorporated into the SFR-SCC mixture. The largest size of the waste glass aggregate measures 9.5 mm, and its water absorption rate is 0.09. The classification and visual characteristics of the waste glass aggregates are outlined in Table 1. Figure 1 illustrates the Physical appearance of the waste glass coarse aggregates (WGCA) employed in this research.

#### Admixture

In this research, in order to achieve self-compacting concrete with optimal performance, a high range water reducer polycarboxylic-based superplasticizer called P10-3R was employed. The specifications of this superplasticizer are in accordance with ASTM C494^68^, as outlined in Table 2.

**Table 2:** Super-plasticizer properties of P10-3R.

Physical properties	Results
Color	Dark green
Density (20 °C)	1.10 ± 0.02
PH value (20 °C)	7.00 ± 1.00
Chlorine (ppm)	500

#### Steel fiber

**Figure 2:**
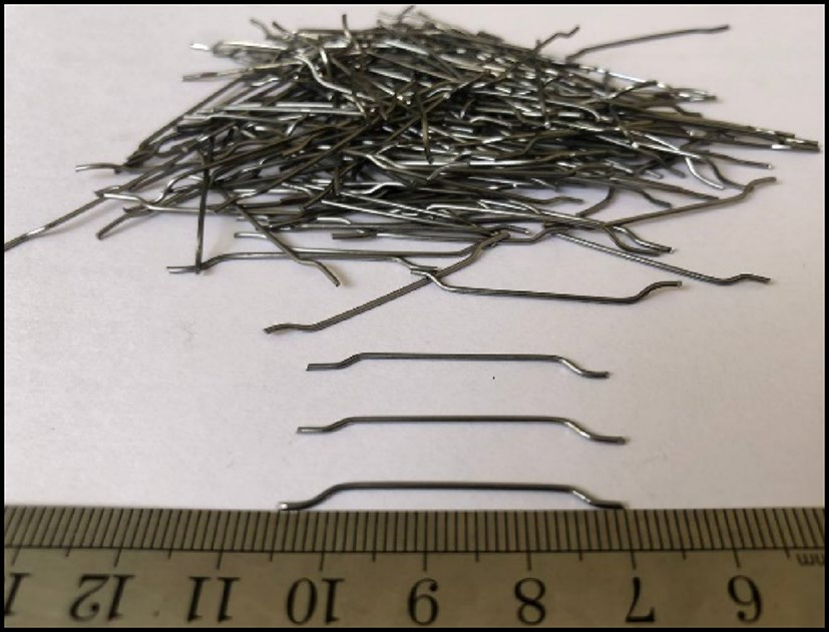
Hooked-end steel fiber.

In this study, steel fibers with a length of 3.5 cm and a diameter of 0.8 mm were incorporated into the SCC mixtures at volume fractions of 0.1, 0.3, and 0.5, as depicted in Fig. 2. This volume fraction is a commonly employed value in various laboratory experiments. As previously mentioned, these fibers were utilized to manage cracking and enhance crack resistance. The specifications of the double hook steel fibers are provided in Table 3.

**Table 3:** Hooked-end steel fiber properties.

Property	Results
Length (mm)	35
Diameter (mm)	0.8
Aspect ratio (L/D)	43.75
Density (Kg/m^3^)	7850
Tensile strength (MPa)	1470
Young's modulus (GPa)	210

### Mix proportions

The study’s SCC mixture proportions was based on EFNARC guidelines^69^. A total of 10 Mix proportions were created to assess new properties, compressive strength, and calculate the final stress intensity factor of modes I and III using ENDB samples. These plans involved replacing natural aggregates with Waste glass coarse aggregates (WGCA) at three different percentages (15%, 30%, and 45%) and adding steel fibers (SF) at varying volume fractions (0.1%, 0.3%, and 0.5%) at ages of 28 and 56 days. The SF was manually added to the rotating mixer to ensure even fiber distribution and minimize the effects of clumping and clustering^70^. This was done to improve the homogeneity of the mixture. Table 4 displays the constituent elements of each concrete mix design separately. Because WGCA has an extremely low absorption rate, HRWR was utilized to minimize the bleeding of the fresh mixture. The purpose of incorporating LSP was to enhance the mixture’s viscosity and segregation resistance^22^.

**Table 4:** Mixture Proportions of SCC comprising WG aggregates and Steel fibers.

Mix ID	Cement (kg/m^3^)	LSP (kg/m^3^)	Water (kg/m^3^)	W/B	Aggregates (kg/m^3^)			SF (kg/m^3^)	SP (l/m^3^)
					Fine	Coarse	WG		
Control	374	122.5	201	0.40	958	898	0	0	4.8
WG15SF0.1	374	122.5	201	0.40	958	763.3	134.7	7.85	4.8
WG15SF0.3	374	122.5	201	0.40	958	763.3	134.7	23.55	4.8
WG15SF0.5	374	122.5	201	0.40	958	763.3	134.7	39.25	4.8
WG30SF0.1	374	122.5	201	0.40	958	628.6	269.4	7.85	4.8
WG30SF0.3	374	122.5	201	0.40	958	628.6	269.4	23.55	4.8
WG30SF0.5	374	122.5	201	0.40	958	628.6	269.4	39.25	4.8
WG45SF0.1	374	122.5	201	0.40	958	493.9	404.1	7.85	4.8
WG45SF0.3	374	122.5	201	0.40	958	493.9	404.1	23.55	4.8
WG45SF0.5	374	122.5	201	0.40	958	493.9	404.1	39.25	4.8

### Casting and curing SCC specimens

**Figure 3:**
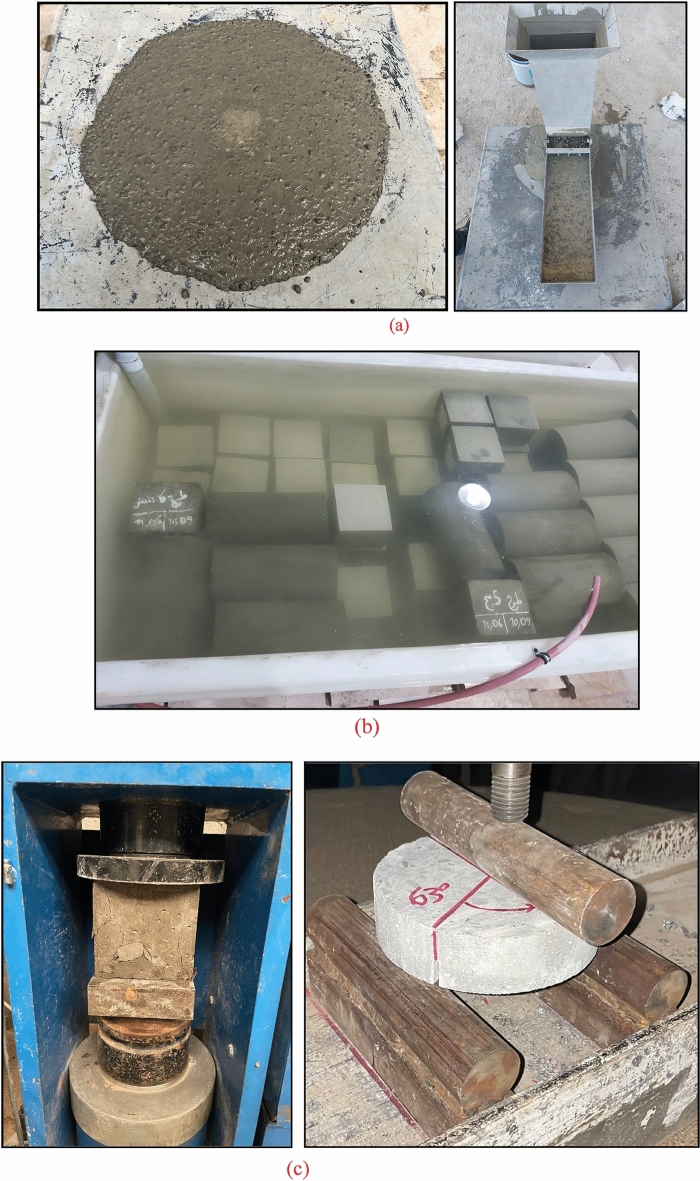
The steps involved in conducting this experimental research include: (**a**) testing fresh concrete, (**b**) curing, and (**c**) testing hardened concrete.

After preparing and weighing the materials, they were added to the mixer in five separate parts. First, add all aggregates and mix them. Then, add fifty percent of the water weight. Next, incorporate the binder materials (cement and limestone powder). Following this, add the remaining water and superplasticizer. Finally, complete the process with the final mixing stage (if incorporating fibers). Each mixing cycle typically lasted about five minutes. After extracting the mixtures from the mixer, the slump flow test is performed before being poured into molds. Two types of molds were utilized in this study: cylindrical molds measuring 150 × 300 mm^2^ and cubic molds measuring 150 × 150 mm^2^. It is worth noting that cylindrical and cubic molds were employed to study fracture parameters and compressive strength, respectively. To assess the compressive strength of each design, 4 cube samples were created, resulting in a total of 40 cube samples for all designs. After 24 h, the concrete samples were removed from their molds and placed in a standard curing tank filled with water at a temperature of 20 ± 3 °C until they reached the desired age for testing, which was either 28 or 56 days. Figure 3 illustrates the sequence of fresh concrete testing, processing, and hardened concrete testing stages.

## Methodology

### Fresh concrete property tests

In accordance with the guidelines established by the European Federation of National Associations representing producers and applicators of specialist products for structures (EFNARC), self-compacting concrete is attained when the concrete mixture exhibits optimal performance concerning fillability and passability, both meeting specified thresholds. The EFNARC guidelines delineating the essential steps to achieve the desired SCC are elaborated in Table 5.

**Table 5:** Criteria for acceptance of self-compacting concrete as per EFNARC standards.

Test method	Unit	Range	
		Minimum	Maximum
Slump flow	mm	550	850
L-Box	–	0.80	1.00

#### Slump flow test

The purpose of this test was to measure the flowability, filling ability, and free deformability of SCC, under the influence of self-weight and absence of any external restriction such as surface friction and obstruction^71^. The test was performed according to the EFNARC standard^72^. A standard slump cone, as described in ASTM C143-12^73^, was used to conduct the test. SCC mixture was poured into the cone without compaction, and the diameter of the resulting circle after lifting the cone was measured. This value, known as the slump flow, is the average of two orthogonal diameters of the circle and was used to evaluate each design.

#### L-Box test

The L-Box test, as defined by EFNARC^72^, is a method used to assess the ability of SCC mixtures to flow through obstacles without losing their cohesion and blocking. The obstacles consist of 2 or 3 bars placed in front of a valve. The vertical part of the box is filled with concrete, and the valve is opened to allow the concrete to flow into the horizontal part. The L-Box blocking ratio is the ratio of the height of the concrete in the horizontal part of the box (H_2_) to the height of the remaining concrete in the vertical part (H_1_). This ratio should be between 0.8 and 1. If the ratio drops below 0.8, it suggests that the viscosity of the concrete mixture is too high, which can cause blockage around the reinforcements.

### Compressive strength test

The compressive strength test is commonly used to assess the quality of concrete and its performance in real-world applications^74,75^. In this study, the uniaxial compressive strength test was performed in accordance with the BS EN 12390-3 standard requirements^76^. Cube samples measuring 150 × 150 mm^2^ were used for this test, which was conducted at both 28 and 56 days of age.

### Preparing SCC disc shape specimens and testing fracture toughness

**Figure 4:**
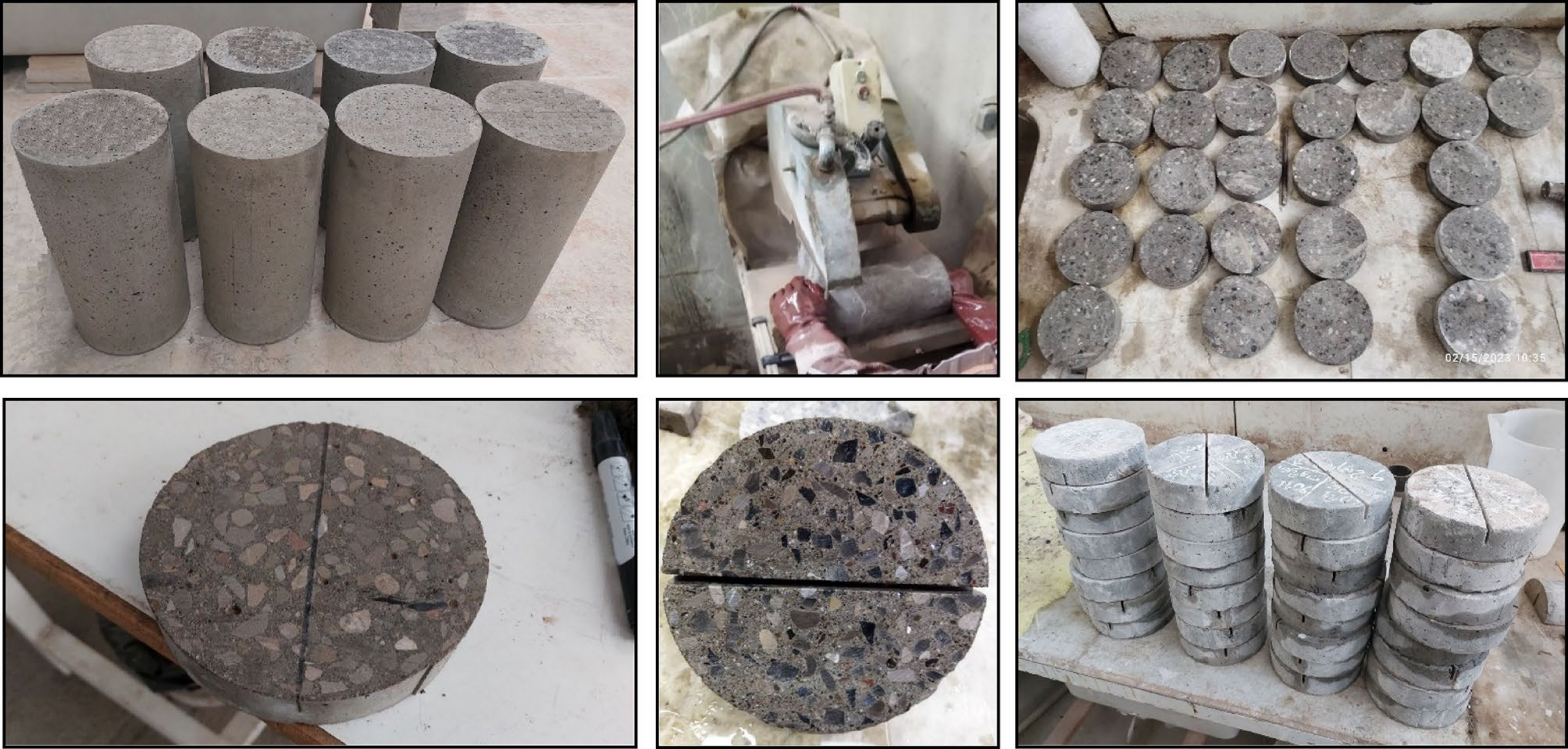
Process of ENDB specimen preparation.

**Figure 5:**
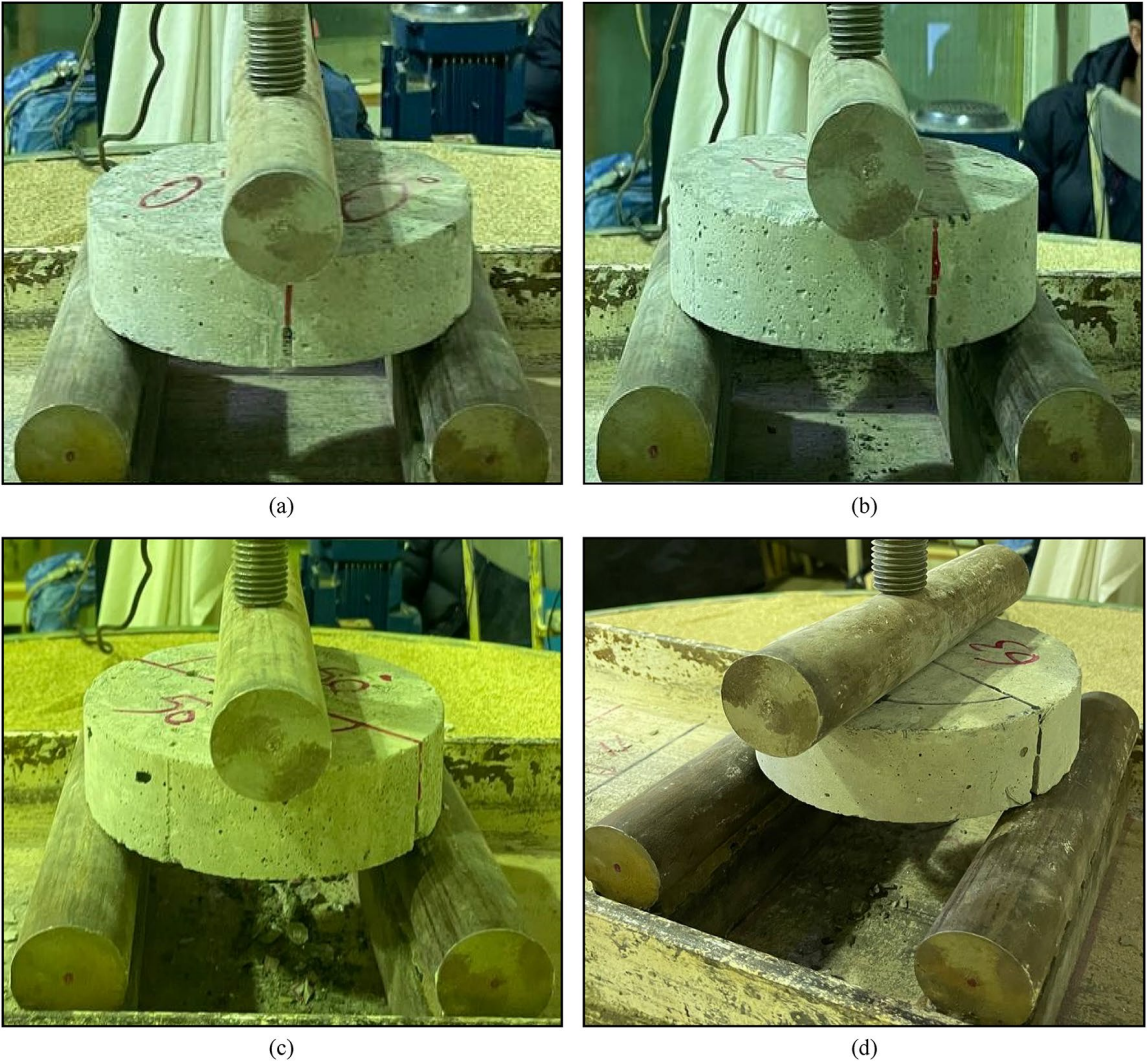
ENDB specimens under various modes that include (**a**) pure mode I, (**b**) mode I/III, (**c**) mode III/I, and (**d**) pure mode III.

**Figure 6:**
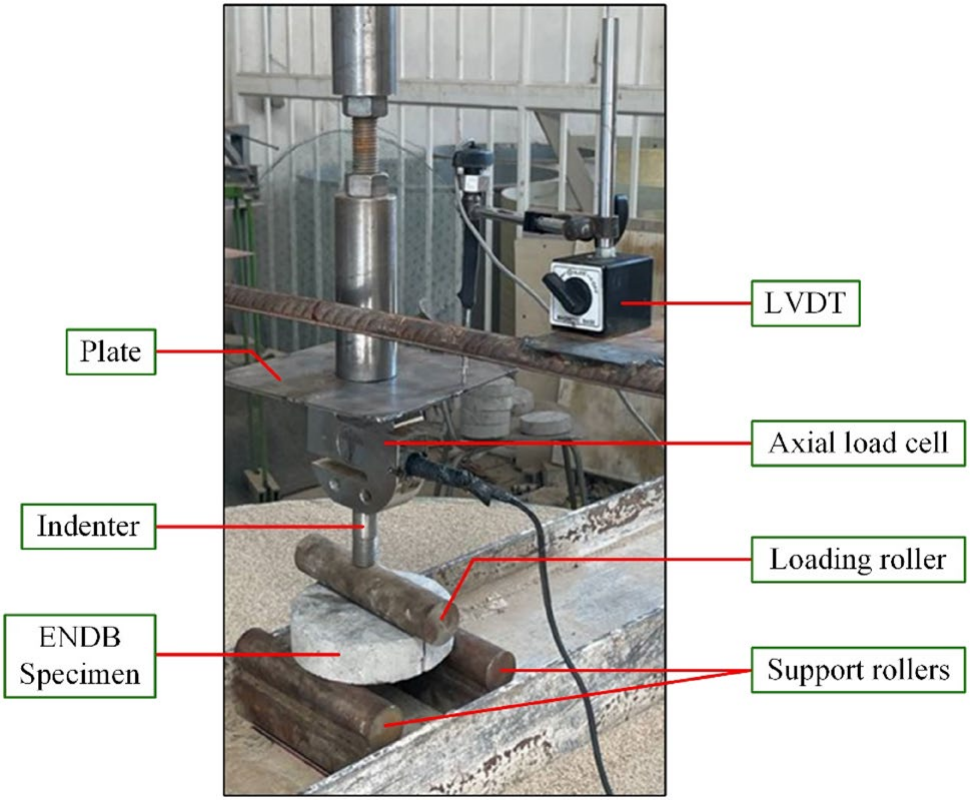
ENDB test set-up.

As previously mentioned, Aliha et al. developed ENDB samples to calculate the critical stress intensity factor in fracture modes I, III, and their mixed modes^56,57^. To prepare these samples, a 150 × 300 mm cylindrical sample is first cut to a thickness of 40 mm. A notch with a height of 16 mm is then made along the diameter of the resulting disc-shaped sample, and the ENDB samples are created, as shown in Fig. 4. The critical load and stress intensity factor of fracture modes I, I/III, III/I, and III are determined using three-point bend loading. The samples are placed on two supports with a distance of 142.5 mm, and the direction of force application is taken into account with deviation angles of 0, 20, 50, and 63 degrees from the crack. For each deviation angle, three samples were created. The loading method for each sample based on the desired deviation angle and fracture mode is illustrated in the Fig. 5. The loading rate for all samples was fixed at 2.1 mm/min. The displacement was measured using an LVDT with $10^{ - 5}$ mm accuracy. The components of the setup for testing are presented in Fig. 6. The critical load (P_Cr_) for each sample was obtained, and fracture toughness parameters, including effective stress intensity coefficients (K_eff_) and critical stress intensity for pure modes I (K_crI_) and III (K_crIII_), were calculated using Eqs. (1–3). Based on the values obtained for K_crI_ and K_crIII_, it was possible to determine the share contributions of loading of each mode I and III loading specified in the considered deviation angles. To accomplish this objective, the complex parameter (M_cl_^e^) was utilized, which has a value ranging from 0 to 1, as shown in Eq. 4. The value of this parameter for pure modes I (M_I_^e^) and III (M_III_^e^) is 0 and 1, respectively.

1 $${\text{K}}_{{cr_{I} }} = \frac{{6P_{cr} S}}{{Rt^{2} }}\sqrt {\pi a} Y_{I}$$

2 $${\text{K}}_{{cr_{III} }} = \frac{{6P_{cr} S}}{{Rt^{2} }}\sqrt {\pi a} Y_{III}$$

3 $${\text{K}}_{eff} = \sqrt {\left( {{\text{K}}_{{cr_{I} }} } \right)^{2} + \left( {{\text{K}}_{{cr_{III} }} } \right)^{2} }$$

4 $${\text{M}}_{cl}^{e} = \frac{2}{\pi }\tan^{ - 1} \left( {\frac{{{\text{K}}_{{cr_{I} }} }}{{{\text{K}}_{{cr_{III} }} }}} \right)$$

The S, R, t, a, Y_I_ and Y_III_ include, respectively, half distance between two supports (71.25 mm), sample radius (75 mm), sample thickness (40 mm), groove height (16 mm), Y_I_ and Y_III_ are the geometric coefficients of the pure modes I and III, which were chosen according to the reference^57^. The values of these geometric coefficients of the modes are listed in Table 6.

**Table 6:** Geometric coefficients related to various loading modes.

Modes	Degree (°C)	Y_I_	Y_III_
Pure-I	0	0.323	0
I/III	20	0.269	0.056
III/I	50	0.1	0.083
Pure-III	63	0	0.073

## Results and discussion

### Fresh concrete properties

#### Slump flow test

**Figure 7:**
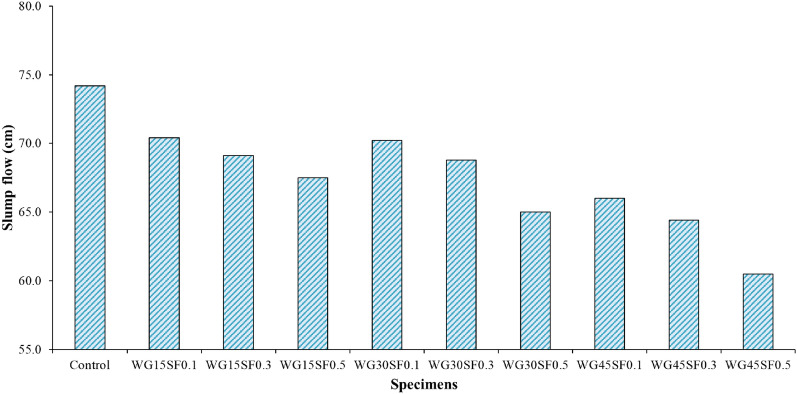
Slump flow test results of SFR-SCC mixtures containing WGCA.

Figure 7 displays that the slump flow value of all samples made is less than that of the control sample, and the range of all slumps is between 60.5 and 74.2 cm. Upon comparing the outcomes of samples containing 0.1%, 0.3%, and 0.5% steel fibers, it was observed that the diameter of the slump flow reduces with an increase in WGCA. This phenomenon is because of a decrease in the weight of the concrete mixture, a smoother surface, and the different configuration of the glass grains. Fathi et al. also arrived at this conclusion^77^. Moreover, by increasing SF, the reduction in the diameter of the slump flow in the samples containing 45% WGCA is more significant than the other samples. The highest and lowest reduction in the slump value of samples containing SF and WGCA compared to the reference sample were related to WG45SF0.5 samples with 18.46% and WG15SF0.1 with 5.12%, respectively. In mixtures containing 15, 30, and 45% WGCA, it was also observed that flowability declined with increasing SF content. In general, adding more SF to the concrete mixture led to a decrease in its slump flow. This is due to the increase in friction between the aggregates and fibers, which makes it harder for the aggregates to roll over each other. According to Turk et al., an increase in the volume fraction of macro steel fibers in the SCC mixture led to a reduction in the amount of slump flow compared to the reference sample^78^.

#### L-Box test

**Figure 8:**
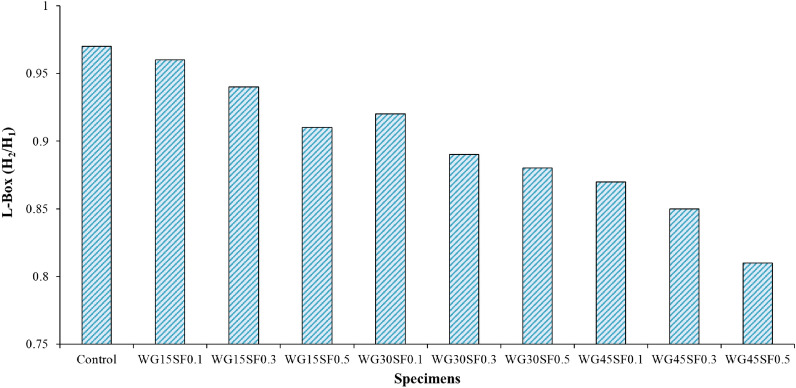
L-Box test results of SFR-SCC mixtures containing WGCA.

Based on the Fig. 8, the L-Box ratio test, using two rebars, results for all mixtures in this study, fall within the range of 0.81–0.97. Similar to the slump flow test findings, the results indicate that all mixtures containing WGCA and SF have lower values than the control sample. This suggests that the reduced flowability results in a decrease in the filling and passing ability. Gencel et al. conducted tests on the workability of SCC containing fly ash and SF with volume fraction percentages of 0.2, 0.4, 0.6, and 0.8 and observed that the fluidity, flow, and passing ability of the mixtures significantly decreased with an increase in the amount of SF^79^. The mixtures with the lowest amount of natural coarse aggregate replacement with WGCA demonstrated better workability when compared to other mixtures. The blockage ratio results for the WG15SF0.1, WG15SF0.3, and WG15SF0.5 samples were 0.96, 0.94, and 0.91, respectively. The study found that as the volume fraction of SF and WGCA increased simultaneously in the mixtures, their passing ability decreased. However, during the experiment, no separation between the components of the mixture was observed. The WG45SF0.5 mixture showed the lowest workability with a value of 0.81. This factor results in a decrease in the adhesion between waste glass coarse aggregate (WGCA) and cement adhesive^43^. Conversely, the lower specific weight of WGCA compared to natural aggregate and the higher stiffness of steel fibers (SF) in comparison to other types of fibers contribute to partial locking and the slowing down of the mixture’s movement when passing through the box valve as these two parameters increase in the mixture. This phenomenon was observed in Ghasemi et al. investigation, which examined the impact of different maximum sizes of natural aggregate (9.5, 12.5, and 19.5) on SCC containing SF with varying volume fractions (0.1%, 0.3%, and 0.5%)^80^. Their study showed that the blocking ratio of the mixtures ranged from 0.81 to 0.9, and increasing the aggregate size reduced the space in the mixture for uniform distribution and proper dispersion of SFs.

#### Evaluating the correlation between Slump flow and L-box test

**Figure 9:**
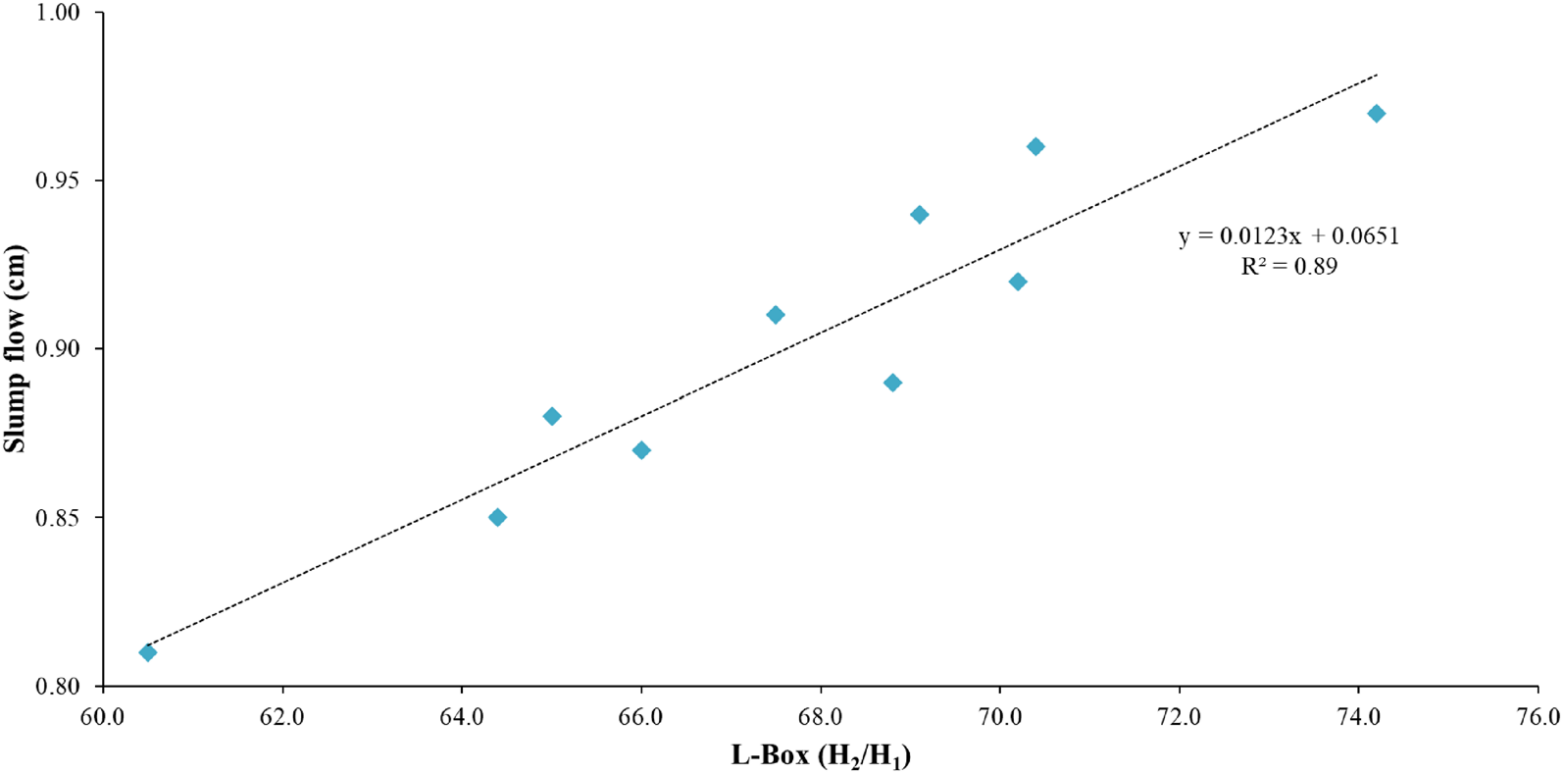
Correlation between results of Slump flow and L-Box tests.

In general, a study of the correlation between parameters affecting the fresh properties of concrete, obtained through tests related to each characteristic, helps to identify the changes that occur in the main constituents of concrete in the mixing stage when other materials are added to the mixture. Figure 9 presented indicates that there is a linear regression relationship between the slump flow and blockage ratio of the mixing design used in this study and R^2^ value for this relationship was found to be 0.89. As previously noted, a reduction in the flowability and fluidity of SCC mixtures results in a decrease in their ability to pass through obstacles in the box. Afshoon et al. evaluated the parameters of fresh SCC containing steel fibers and copper slag aggregate and reported a good linear correlation between the results of the slump flow diameter test with L-Box. The R^2^ value for this relationship was 0.88^75^.

### Hardened concrete properties

#### Compressive strength test

**Figure 10:**
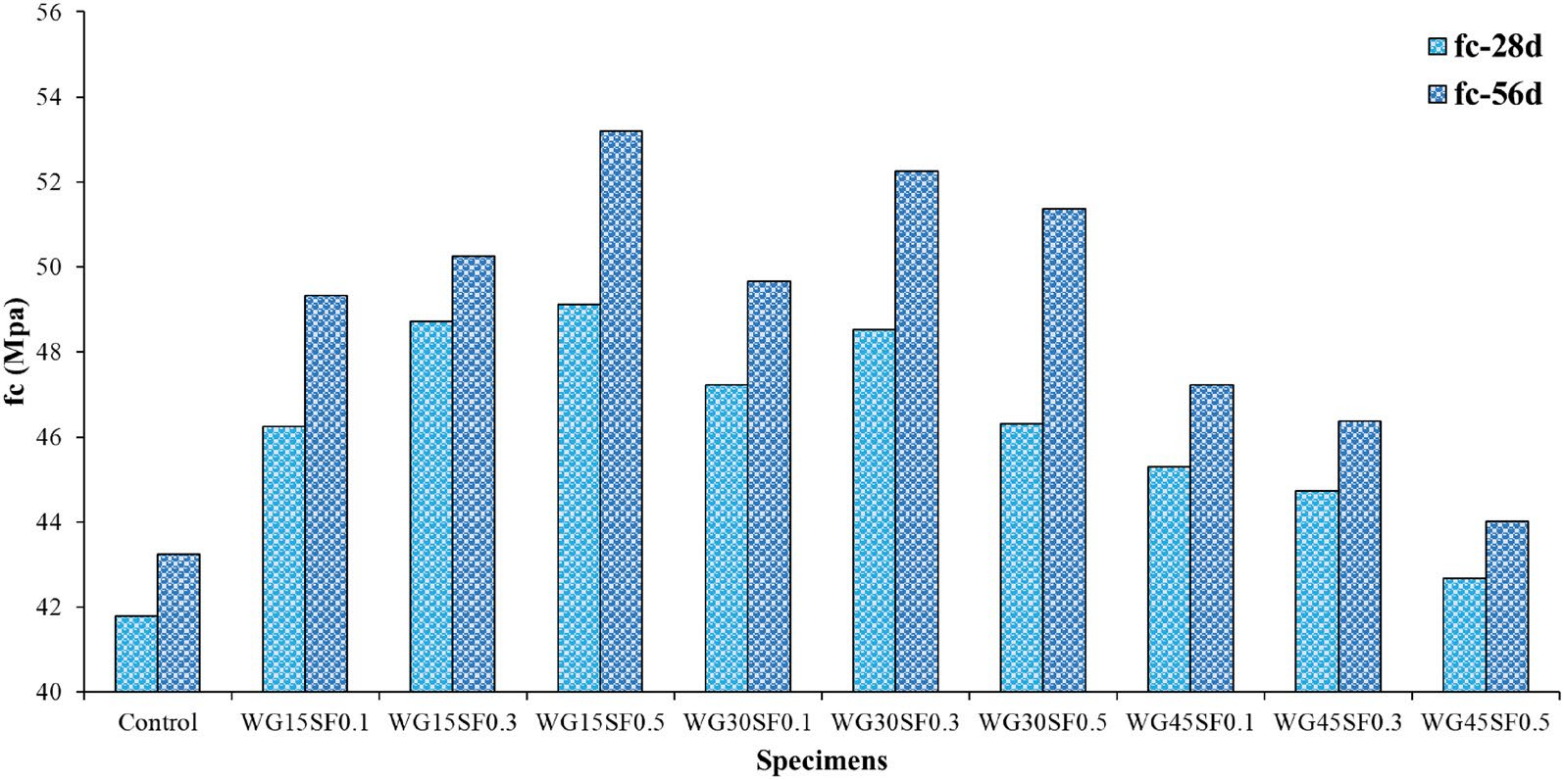
Compressive strength test of SFR-SCC specimens containing WGCA at 28 and 56 ages.

The Fig. 10 illustrates the results of the uniaxial compressive strength test conducted on cubic samples (measuring 150 × 150 mm) after 28 and 56 days. The compressive strength values of all samples on both days were higher than the control sample. Samples containing 15% and 30% WGCA, in comparison to those containing 45% WGCA, exhibited better flowability and passing ability, as well as higher compressive strength values at 28 and 56 days. The addition of WGCA to SCC mixtures resulted in greater limitation of compressive strength at both processing ages, when compared to the addition of SF. In a study conducted by Fathi et al., it was observed that increasing both GFA and polypropylene fibers in SCC samples simultaneously resulted in a greater reduction in strength due to GFA than fibers^77^. The compressive strength of SCC containing different percentages of GFA (30%, 50%, 70%, and 100%) and polypropylene fibers (0.5%, 1%, and 1.5%) was investigated in this study. Kou and Poon assessed the impact of replacing fine and coarse natural aggregates with waste glass on SCC and found that the compressive strength of all samples decreased compared to the control design^43^. The cause of reduced compressive strength in concrete samples containing glass waste is due to the shape of the waste glass aggregates, its low resistance compared to natural aggregate, its low water absorption, and its inability to participate in the ASR reaction between aggregate and cement. This results in poor bonding between the glass aggregate and cement matrix, leading to the formation of holes and microcracks at the paste/aggregate interface area and ultimately weakening the concrete. However, increasing the values of waste glass and SF simultaneously in the design can increase the compressive strength of concrete samples. Fathi et al. explained that this increase in strength is due to the cubic samples being under both pressure and tension in two perpendicular directions when placed in the uniaxial compression jack device^77^. The fibers in the concrete play a crucial role in preventing crack growth, increasing modulus of rupture and tensile strength, and ultimately increasing the compressive strength of the cubic samples. Fathi et al. also noted that diagonal cracks appeared on the surface of the cubic samples after the uniaxial compression test^77^. In studies conducted by Nematzadeh et al., it was reported that increasing the steel fiber content in concrete, which includes zeolite and recycled nylon granules, enhances impact energy, mitigates the induced reduction, improves mechanical properties, and enhances the ultimate deflection capacity of both heated and non-heated concretes^81,82^.

The compressive strength of the reference sample at the age of 28 days is 41.78 MPa and the other samples are in the range of 42.67–49.12 MPa. The highest and lowest growth in the compressive strength of concrete samples containing WGCA and SF at this age compared to the control sample were related to WG15SF0.5 and WG45SF0.5 samples with values of 17.57 and 2.13%, respectively. The addition of SF to the 28-day samples containing WGCA improved the compressive strength of all samples compared to the control design. This can be attributed to the fact that fibers enhance the strength against applied load by reducing stress concentration, creating bridge cracks, and delaying the propagation and growth of macro-cracks. In a study assessing the impact of SF and synthetic fibers on concrete, Buratti et al. reported that SFs performed better than other fibers in controlling and propagating cracks due to their harder nature when the same amounts of fibers were passed through a crack propagation plane^21^.

As the curing age of the samples raised to 56 days, the compressive strength of all samples, particularly those containing WGCA, raised compared to their 28-day strength. The WG30SF0.5 sample exhibited the highest ratio increase in compressive strength compared to its 28-day strength with a value of 10.9%, although this rising was less than the previous one. The reason for this enhancement in designs containing WGCA compared to their 28-day strength is due to the low water absorption of glass and the presence of free water within the mixture, which reduces drying shrinkage of concrete samples and ultimately delays the completion of the cement hydration reaction. Kou and Poon also recognized that an increase in the fine and coarse grain of glass leads to a decrease in the shrinkage of SCC samples^43^. Based on the Fig. 10, the 56-day compressive strength of the control design is 43.25 MPa, while the values for the other samples fall within the range of 44.02–53.2 MPa. Escalating the amount of SF in samples containing 15% and 30% WGCA generally led to a growth in compressive strength, except for the WG30SF0.5 sample. However, for the samples with 45% replacement, increasing the amount of SF resulted in a decrease in compressive strength. The reason for this decline in strength is due to an increase in holes, micro-cracks, and capillary openings in the interfacial transition cement/aggregates zone.

#### Fracture toughness test

**Figure 11:**
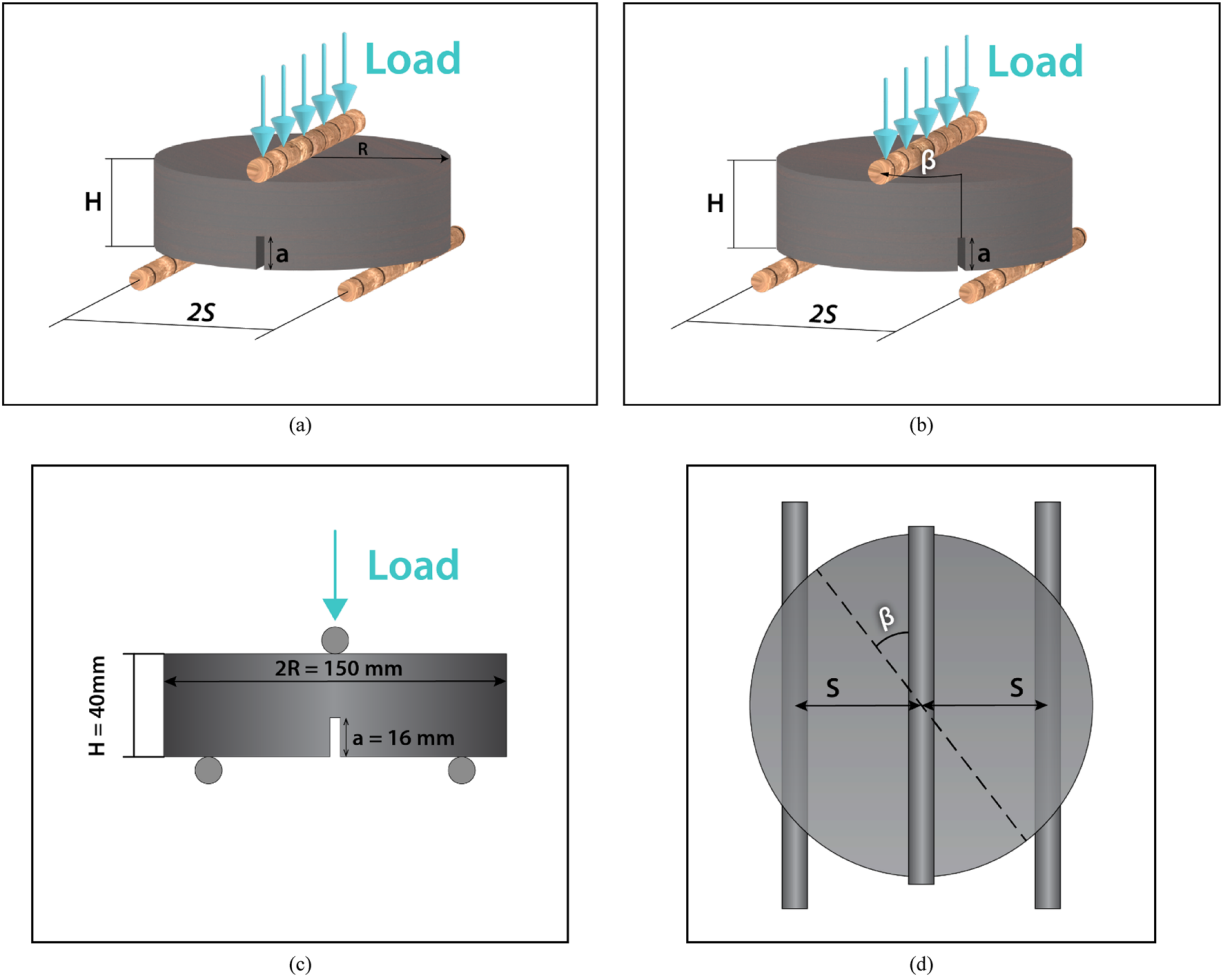
Geometry configuration and loading condition of ENBD specimen employed for determining pure mode I and other modes (I/III, III/I, and III) fracture toughness study.

**Figure 12:**
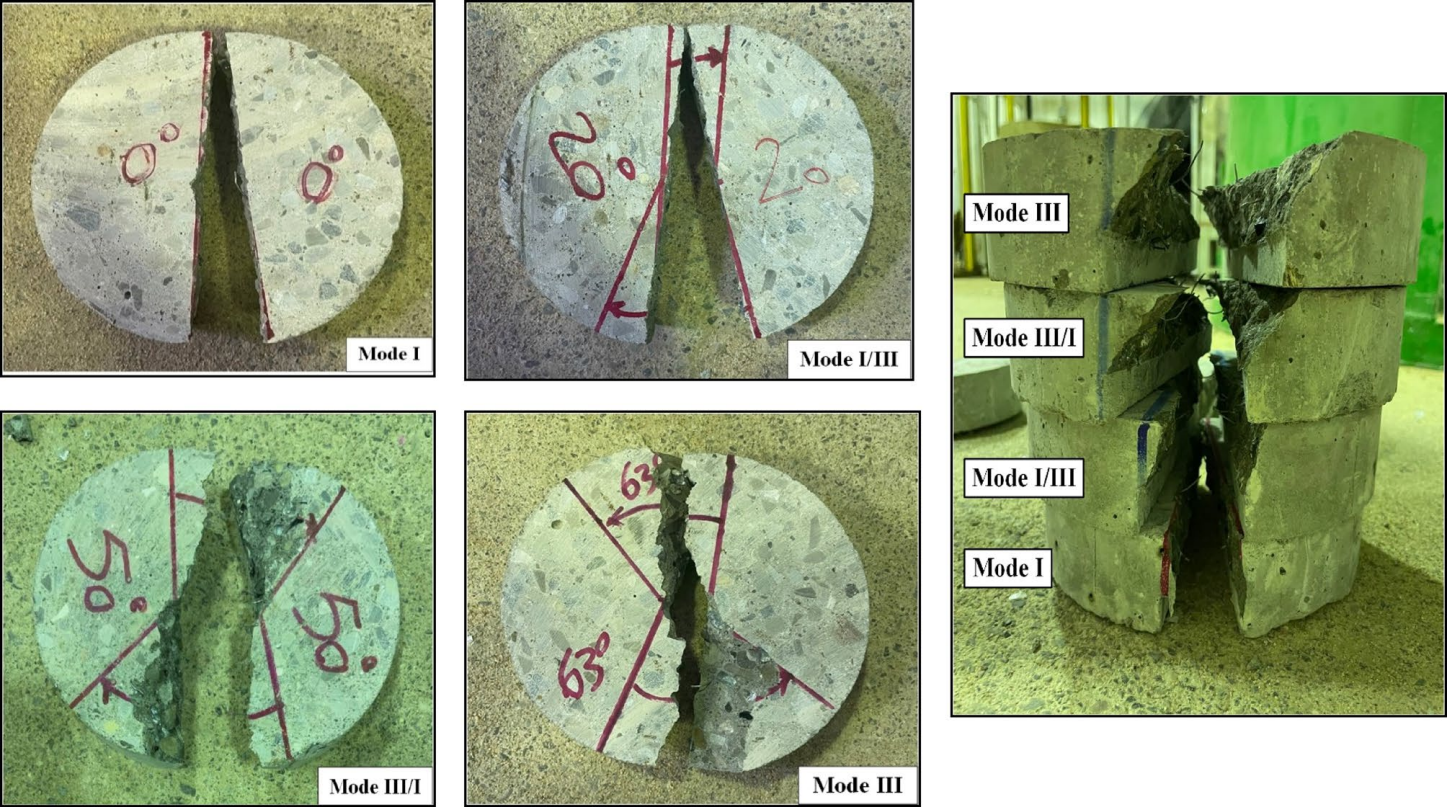
Failure path patterns of ENDB specimens under different loading modes.

After setting the ENDB samples under the device based on the failure mode, loading was applied. Figure 11 shows the loading settings included to evaluate the fracture toughness parameters of SFR-SCC samples containing WGCA and the control sample in pure modes I, I/III, III/I and pure III. Figure 12 illustrates the failure of WG30SF0.5 samples under loading in different modes. The figure demonstrates that the specimen subjected to mode I (β = 0°) is nearly divided into two equal parts. However, as the deviation angle increases to 63°, the fracture pattern path deviates significantly from the primary notch. Table 7 presents the average fracture toughness test results obtained from SFR-SCC samples containing waste glass coarse aggregates (WGCA) and the reference sample at 28 and 56 days of age, categorized by failure modes (III, III/I, I/III, and I). Subsequently, the factors influencing the results of the fracture toughness parameters of the samples are examined individually.

**Table 7:** Results of the fracture toughness test

Specimens	Age	Mode I					Mode I/III					Mode III/I					Mode III				
		β = 0°					β = 20°					β = 50°					β = 63°				
		${\text{P}}_{{{\text{Cr}}}}$	${\text{K}}_{{{\text{cr}}_{{\text{I}}} }}$	${\text{K}}_{{{\text{cr}}_{{{\text{III}}}} }}$	${\text{K}}_{{{\text{eff}}}}$	${\text{M}}_{{{\text{cl}}}}^{{\text{e}}}$	${\text{P}}_{{{\text{cr}}}}$	${\text{K}}_{{{\text{cr}}_{{\text{I}}} }}$	${\text{K}}_{{{\text{cr}}_{{{\text{III}}}} }}$	${\text{K}}_{{{\text{eff}}}}$	${\text{M}}_{{{\text{cl}}}}^{{\text{e}}}$	${\text{P}}_{{{\text{Cr}}}}$	${\text{K}}_{{{\text{cr}}_{{\text{I}}} }}$	${\text{K}}_{{{\text{cr}}_{{{\text{III}}}} }}$	${\text{K}}_{{{\text{eff}}}}$	${\text{M}}_{{{\text{cl}}}}^{{\text{e}}}$	${\text{P}}_{{{\text{Cr}}}}$	${\text{K}}_{{{\text{cr}}_{{\text{I}}} }}$	${\text{K}}_{{{\text{cr}}_{{{\text{III}}}} }}$	${\text{K}}_{{{\text{eff}}}}$	${\text{M}}_{{{\text{cl}}}}^{{\text{e}}}$
Control	28	1635.60	0.42	0.00	0.42	1.00	2136.41	0.46	0.10	0.47	0.86	3751.22	0.30	0.25	0.39	0.56	4787.92	0.00	0.28	0.28	0.00
	56	1815.19	0.47	0.00	0.47	1.0	2353.68	0.51	0.11	0.52	0.86	3996.32	0.32	0.26	0.41	0.57	5086.92	0.00	0.30	0.30	0.00
WG15SF0.1	28	1840.05	0.47	0.00	0.47	1.00	2385.23	0.51	0.11	0.52	0.86	4004.65	0.32	0.27	0.42	0.55	5081.78	0.00	0.30	0.30	0.00
	56	2076.24	0.54	0.00	0.54	1.00	2617.62	0.56	0.12	0.57	0.87	4253.49	0.34	0.28	0.44	0.56	5337.57	0.00	0.31	0.31	0.00
WG15SF0.3	28	1998.48	0.52	0.00	0.52	1.00	2523.85	0.54	0.11	0.55	0.87	4138.91	0.33	0.27	0.43	0.56	5211.20	0.00	0.30	0.30	0.00
	56	2183.07	0.56	0.00	0.56	1.00	2724.73	0.59	0.12	0.60	0.87	4358.14	0.35	0.29	0.45	0.56	5432.41	0.00	0.32	0.32	0.00
WG15SF0.5	28	2085.75	0.54	0.00	0.54	1.00	2618.68	0.56	0.12	0.57	0.87	4261.49	0.34	0.28	0.44	0.56	5347.08	0.00	0.31	0.31	0.00
	56	2294.83	0.59	0.00	0.59	1.00	2837.48	0.61	0.13	0.62	0.87	4470.17	0.36	0.30	0.46	0.56	5554.79	0.00	0.32	0.32	0.00
WG30SF0.1	28	2148.52	0.55	0.00	0.55	1.00	2687.11	0.58	0.12	0.59	0.87	4326.18	0.35	0.29	0.45	0.56	5403.87	0.00	0.32	0.32	0.00
	56	2379.42	0.61	0.00	0.61	1.00	2925.52	0.63	0.13	0.64	0.87	4553.68	0.36	0.30	0.47	0.56	5627.21	0.00	0.33	0.33	0.00
WG30SF0.3	28	2286.42	0.59	0.00	0.59	1.00	2823.17	0.61	0.13	0.62	0.87	4457.93	0.36	0.30	0.46	0.56	5528.59	0.00	0.32	0.32	0.00
	56	2465.54	0.64	0.00	0.64	1.00	3001.95	0.64	0.13	0.66	0.87	4635.29	0.37	0.31	0.48	0.56	5719.72	0.00	0.33	0.33	0.00
WG30SF0.5	28	2376.15	0.61	0.00	0.61	1.00	2918.48	0.63	0.13	0.64	0.87	4543.74	0.36	0.30	0.47	0.56	5621.29	0.00	0.33	0.33	0.00
	56	2574.69	0.66	0.00	0.66	1.00	3127.28	0.67	0.14	0.69	0.87	4768.98	0.38	0.32	0.50	0.55	5852.83	0.00	0.34	0.34	0.00
WG45SF0.1	28	2128.05	0.55	0.00	0.55	1.00	2604.79	0.56	0.12	0.57	0.87	4227.21	0.34	0.28	0.44	0.56	5296.70	0.00	0.31	0.31	0.00
	56	2214.59	0.57	0.00	0.57	1.00	2751.14	0.59	0.12	0.60	0.87	4383.51	0.35	0.29	0.46	0.56	5458.49	0.00	0.32	0.32	0.00
WG45SF0.3	28	2059.94	0.53	0.00	0.53	1.00	2692.89	0.58	0.12	0.59	0.87	4312.01	0.34	0.29	0.45	0.55	5209.71	0.00	0.30	0.30	0.00
	56	2361.27	0.61	0.00	0.61	1.00	2892.05	0.62	0.13	0.63	0.87	4513.77	0.36	0.30	0.47	0.56	5587.14	0.00	0.33	0.33	0.00
WG45SF0.5	28	2001.34	0.52	0.00	0.52	1.00	2464.51	0.53	0.11	0.54	0.87	4178.43	0.33	0.28	0.43	0.55	5035.18	0.00	0.29	0.29	0.00
	56	2119.78	0.55	0.00	0.55	1.00	2661.37	0.57	0.12	0.58	0.87	4487.42	0.36	0.30	0.47	0.56	5351.38	0.00	0.31	0.31	0.00

[Age (Days), ${\text{P}}_{{{\text{Cr}}}}$ (N), ${\text{M}}_{{{\text{cl}}}}^{{\text{e}}}$ (Rad), and ${\text{K}}_{{cr_{I} }}$, ${\text{K}}_{{cr_{III} }}$, ${\text{K}}_{eff}$ (MPa)].

##### Influence of steel fibers

**Figure 13:**
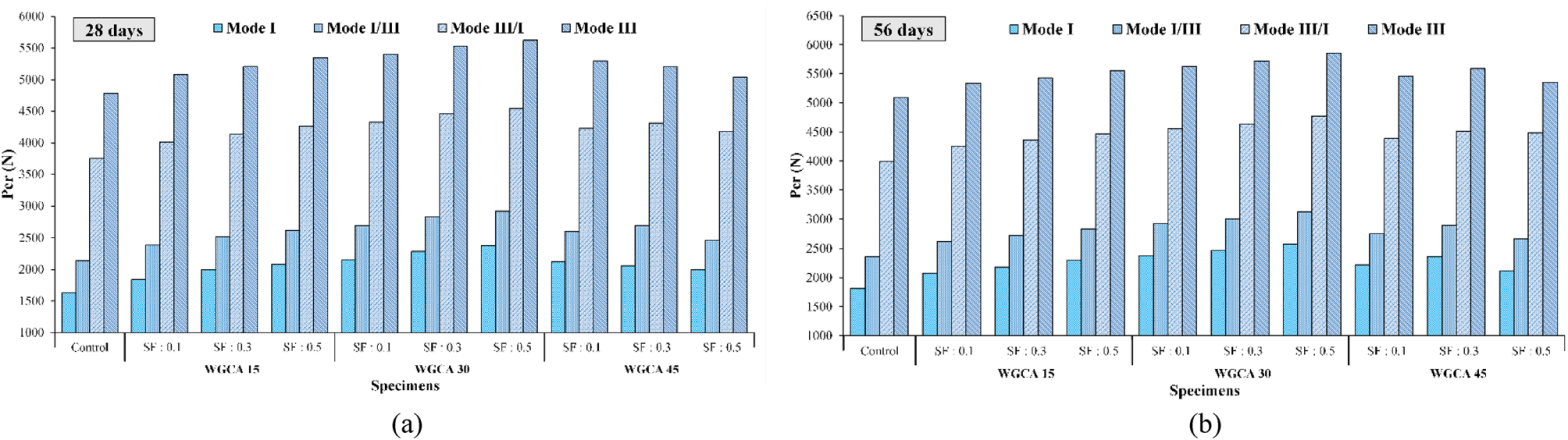
Influence of various SF volume fractions on the critical failure load amounts of different modes of each WGCA volume for ages (**a**) 28 and (**b**) 56 days.

Figure 13 provides the critical load (P_Cr_) values for different modes of SFR-SCC samples containing WGCA, as well as the control sample at 28 and 56 days of age, based on the percentage of WGCA replacement (15%, 30%, and 45%). Based on the figure the lowest and highest values of critical load occurred in pure mode I and pure mode III, respectively, for all samples at both ages. Additionally, it was observed that as the deviation angle increased from 0 to 63 degrees, the P_Cr_ value of each sample escalated significantly compared to the initial cut. For all four considered modes, the P_Cr_ values of all samples were higher than the reference sample at both processing ages. In a study examining the fracture parameters of concrete samples containing recycled asphalt pavement using ENDB forms in modes I and III, Mansourian et al. found that the samples exhibited the highest and lowest values of critical load in pure modes III and I, respectively^83^. Mousavi et al. reported the same results^44^.

According to Fig. 13, the highest value of P_Cr_ among all four modes of all samples, at both ages, corresponds to the WG30SF0.5 sample. This sample has increased compared to the P_Cr_ value of four modes (I, I/III, III/I and III) of the control sample, which includes 45.27, 36.61, 21.13 and 17.41% in 28 days and 41.84, 32.86, 19.33 and 15.05% in 56 days, respectively. The lowest increase in P_Cr_ in 28-day samples compared to their corresponding control samples in modes I, I/III and III/I belongs to the WG15SF0.1 sample with 12.5, 11.64 and 6.75%, respectively, and in mode III corresponding to the WG45SF0.1 sample with a percentage of 5.16. In the 56-day samples, the lowest value of the critical failure load in all four modes corresponds to the WG15SF0.1 sample. According to Fig. 13, with the increase in SF volume fraction percentage from 0.1 to 0.5, the value of P_Cr_ in all modes of samples containing 15 and 30% WGCA replacement showed an upward trend compared to the control sample, in both processing ages. These results revealed that in addition to forming a good bond with the cement matrix, SFs did not show weakness in their main role of reducing the stress concentration point and creating bridge-cracks. For this reason, these samples resisted more loads. Afshoon et al. study on SCC containing SF (0.1, 0.3, and 0.5%) and copper slag aggregate (CSA) with a replacement percentage of 20–60%^84^. They reported that the increase of SF and CSA in the samples improves the fracture energy, flexural strength and flexural toughness. In a manner analogous to this discovery, Madandoust et al. recognized that augmenting the volume fraction of SF leads to an enhancement in the modulus of rupture within the SCC sample^85^. Conversely, the impact of increasing SF content in samples containing 45% WGCA resulted in a reduction in their P_Cr_ value as compared to the 30% replacement, albeit still showing an increase when compared to the 15% replacement. This occurrence can be attributed to the weakened ITZ resulting from an increase in voids in these specimens, leading to a decrease in the effectiveness of fibers in governing the expansion and progression of cracks.

**Figure 14:**
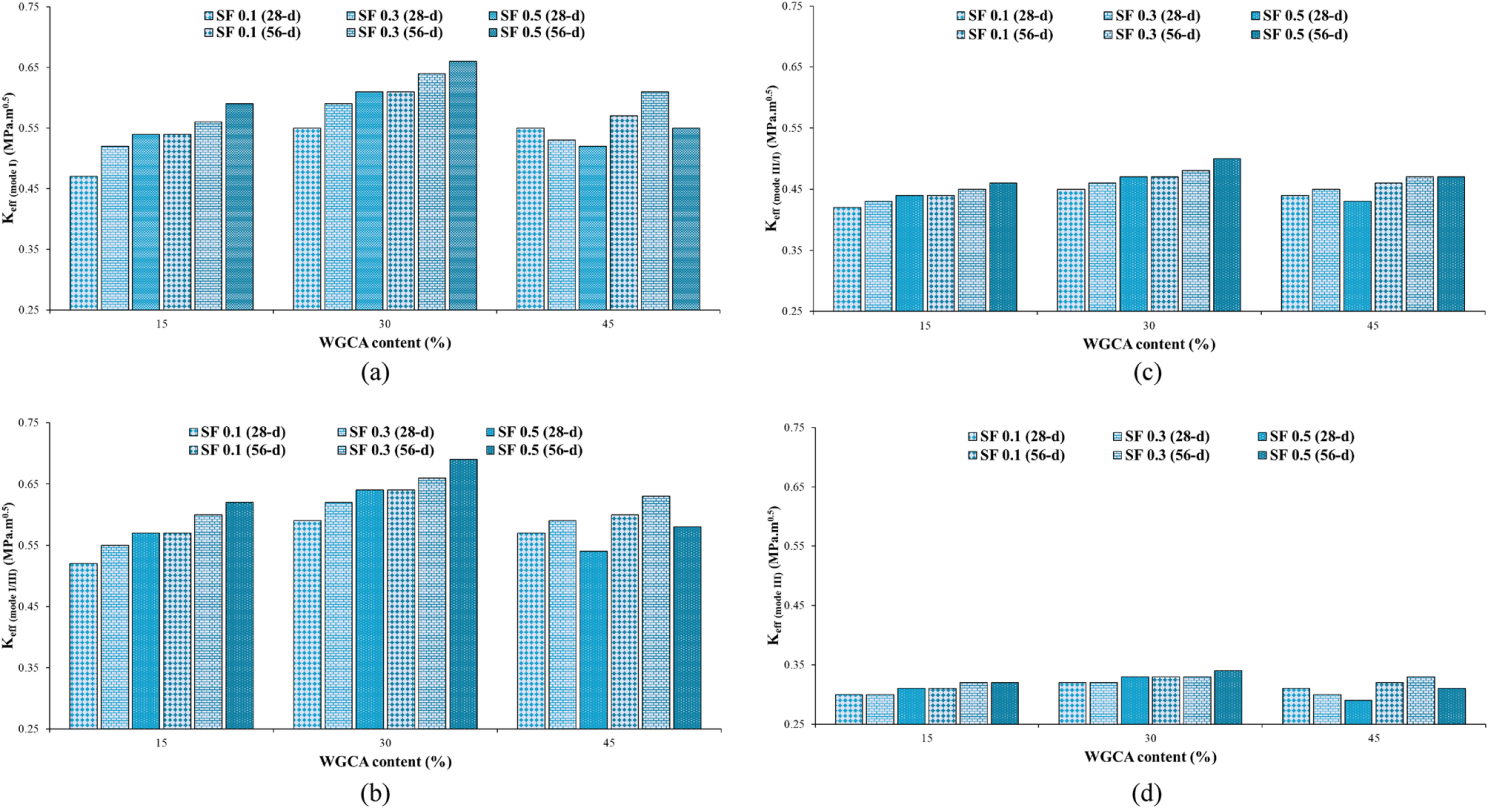
Influence of different SF volume fractions on ${\text{K}}_{{{\text{eff}}}}$ of each WGCA volume for (**a**) pure mode I, (**b**) mode I/III, (**c**) mode III/I, and (**d**) pure mode III.

To analyze the impact of SF on the fracture toughness of the specimens, they were categorized based on the percentages of WGCA replacement (15, 30, and 45%), as demonstrated in Fig. 14. As outlined in Table 7, the K_eff_ values for all samples at both 28 and 56 days, incorporating both SF and WGCA across all four conditions, surpassed those of the control sample. Within the WGCA-containing samples, an escalation in SF content led to an increase in K_eff_ values across all age periods and modes, in comparison with the control sample. However, the extent of this increase varied among different samples. The most substantial rate of K_eff_ enhancement among all modes and ages, when compared to the control sample, was observed in the WG30SF0.5 sample. The most modest augmentation in fracture toughness at the 28-day mark is observed in two samples: WG45SF0.5 in mode III and WG15SF0.1 in modes I, I/III, and III/I. This outcome aligns with the previously discussed fiber crack-bridging effect, indicating that the presence of SF in the specimens improves the material's fracture behavior, rendering it more adaptable compared to the reference sample. Among all the K_eff_ values for distinct modes, the extremes are evident in pure mode III and the combined mode I/III, as depicted in Fig. 14. In a related analysis of fracture parameters for concrete containing reclaimed asphalt pavement (RAP) using ENDB molds, Mansourian et al. similarly noted that the lowest K_eff_ value among the samples was associated with out-of-plane sliding deformation, specifically pure mode III^83^.

**Figure 15:**
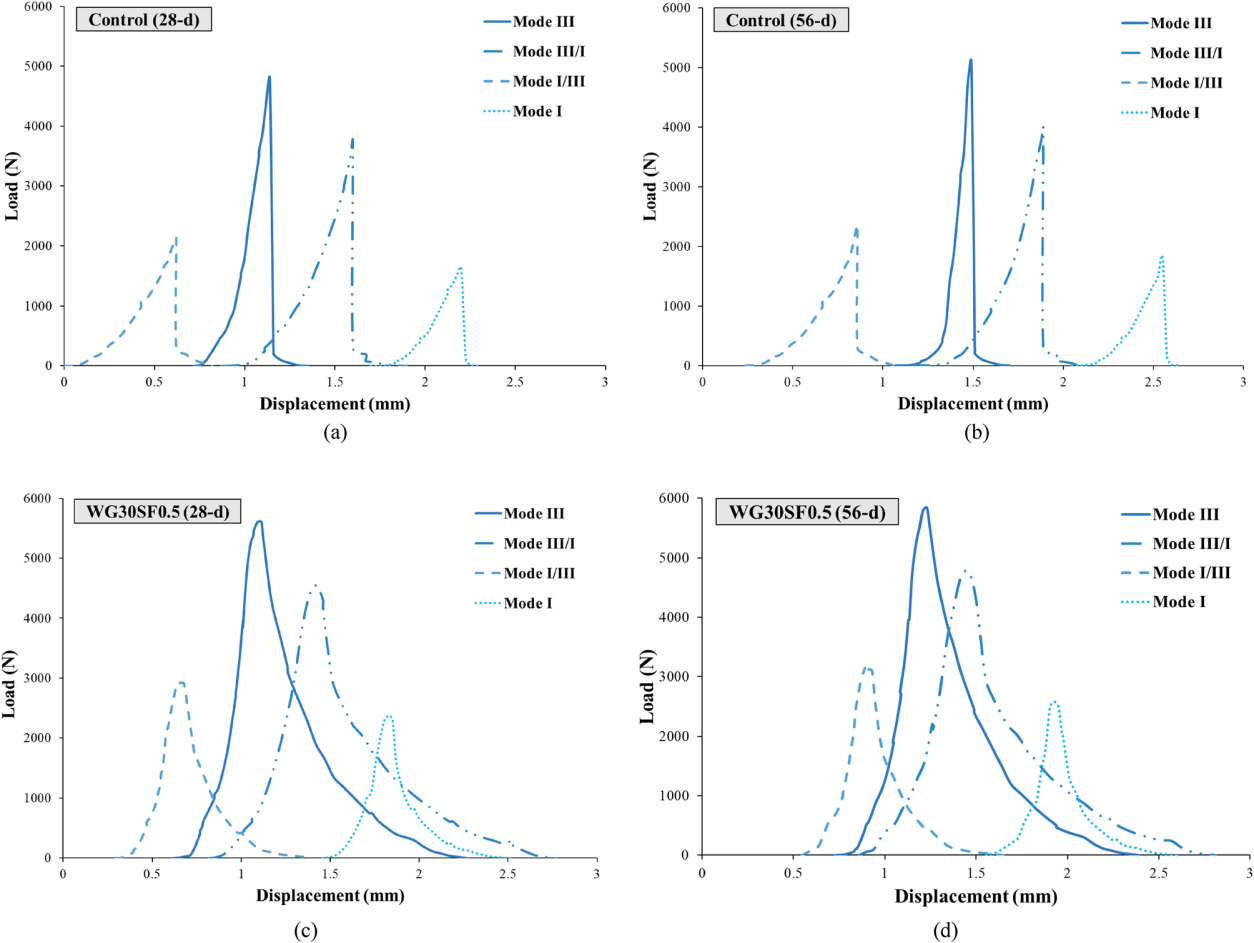
Comparing the load–displacement curves of Control and WG30SF0.5 Specimens (28-d and 56-d) under different loading modes.

Figure 15 shows the force–displacement curve of different modes of control and WG30SF0.5 samples at the ages of 28 and 56 days. In the control sample, when the loading force reaches its maximum value, in the control sample, a sudden (linear) drop occurs in the trend of the force value. However, the behavior of the WG30SF0.5 sample after reaching the critical failure load value was nonlinear. Due to this type of behavior, this sample undergoes more displacement than the control sample until complete destruction. The inclusion of fibers in the specimen also introduces a delay in crack growth and progression, ultimately enhancing the sample's performance post-cracking. Furthermore, aside from the fiber presence, the contribution of WGCA must also be acknowledged. With an optimal percentage, WGCA effectively establishes an interlocking and interaction among the hardened concrete components, thereby boosting the load-bearing capacity in this sample when compared to the control specimen. This particular kind of concrete holds potential for application in structures where there are constraints on the weight of structural components and a desire to enhance load-bearing capabilities. Notably, aside from curbing rebar usage and reducing costs, this concrete variation contributes to the structural effectiveness when faced with dynamic loads those resulting from seismic loads. In a study exploring how variations in the volume fraction of hooked-end steel fibers impact the mechanical attributes of self-compacting concrete, Khaloo et al. indicated that an escalation in fiber content results in improved flexural strength, flexural toughness, absorbed energy, and deflection. These combined factors contribute to rendering concrete elements more adaptable and shapeable^86^.

##### Influence of waste glass coarse aggregates

**Figure 16:**
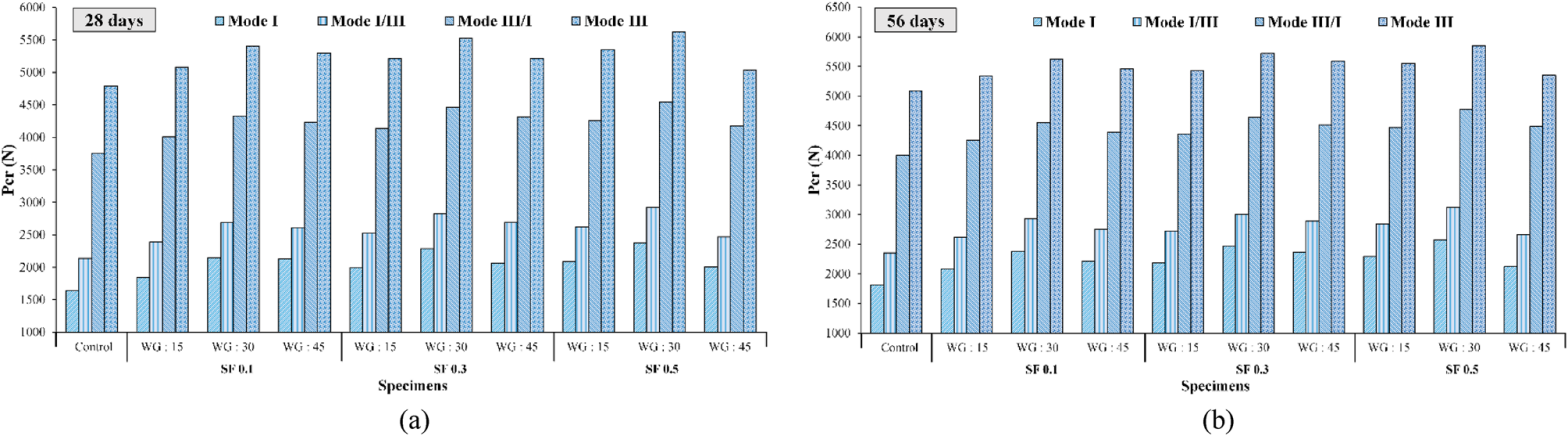
Influence of various WGCA volumes on the critical failure load amounts of different modes of each SF volume fraction for ages (**a**) 28 and (**b**) 56 days.

Figure 16 illustrates the ${\text{P}}_{{{\text{Cr}}}}$ outcomes for concrete samples featuring various volume fractions of SF at replacement percentages of 15, 30, and 45% for waste glass (WG), with aging periods of 28 and 56 days, across different modes. Generally, the incorporation of WGCA instead of natural coarse aggregates in samples containing fibers led to an elevation in their ${\text{P}}_{{{\text{Cr}}}}$ values across all modes and ages. This observation suggests that the bonding between WGCA and the constituents of the concrete matrix, as well as the aggregates/fiber interaction, has been effectively achieved, to the extent that its performance even surpasses that of the control sample. Increasing WGCA replacement up to 45% resulted in a reduction in the ${\text{P}}_{{{\text{Cr}}}}$ value compared to the 30% replacement level in the samples. Specifically, the lowest increase in ${\text{P}}_{{{\text{Cr}}}}$ value within the pure mode III occurred in the sample containing 0.5% SF and 45% WGCA, among all 28-day samples featuring both fibers and glass. This phenomenon arises due to the glass's low water absorption and its non-participation in ASR. Consequently, as the glass content increases, this factor exacerbates, causing a decline in component adhesion and sample strength. However, the presence of fibers in this specific proportion of substitution in the mixtures, encompassing 0.1%, 0.3%, and 0.5% fibers, curbed further ${\text{P}}_{{{\text{Cr}}}}$ reduction. As a result, in the WG45SF0.5 sample, the ${\text{P}}_{{{\text{Cr}}}}$ value across different modes (I, I/III, III/I, and III) increased in comparison to the control sample at both 28 and 56 days. The increments were 22.36%, 15.35%, 11.38%, and 5.16%, respectively, for the 28-day samples, and 16.74%, 13.07%, 12.28%, and 5.19% for the 56-day samples. An examination of the effect of 36 mm-length SF double-headed hooks on concrete containing 20% waste glass particles at the age of 14 days, as conducted by Park and Lee, revealed that with an increase in fiber volume to 1.5%, flexural strength saw a 10% enhancement compared to the reference sample^87^.

**Figure 17:**
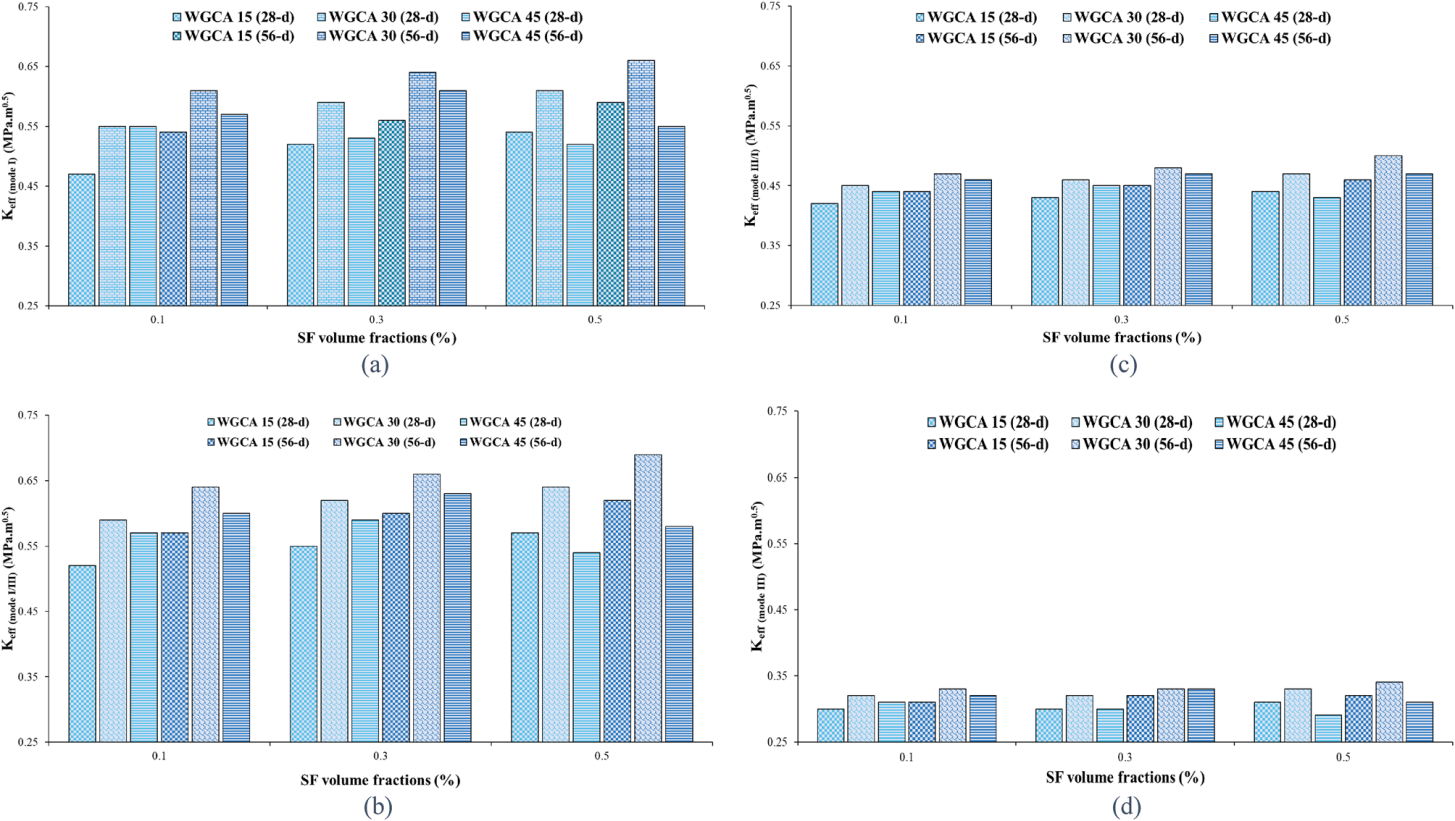
Influence of different WGCA volumes on $K_{eff}$ of each SF volume fraction for (**a**) pure mode I, (**b**) mode I/III, (**c**) mode III/I, and (**d**) pure mode III.

Figure 17 demonstrates the effective stress intensity coefficient values of SCC samples containing WGCA in various modes (pure modes I and III and their combination modes) at different volume fractions of steel fibers (0.1%, 0.3%, and 0.5%). It is observed that as the amount of WGCA increases up to 30% in both 28-day and 56-day concrete samples containing steel fibers (SF), the K_eff_ values show an upward trend. However, when the replacement percentage of WGCA reaches 45%, a decrease in the K_eff_ values is observed in different modes compared to the previous replacement percentage. Notably, exceptions to this trend are observed in the pure mode I of the 28-day sample WG45SF0.1 and the pure mode III of the 56-day sample WG45SF0.3, where no significant change is observed. A similar trend is observed in the P_Cr_ values of the corresponding samples. This can be attributed to the fact that fracture toughness is influenced by the critical fracture load. The reason behind this behavior in samples containing 45% WGCA lies in the structural characteristics of glass, which possesses a smooth, angular, and brittle surface. Additionally, the absorption of water from the concrete matrix results in the accumulation of free water within the glass. Subsequently, as this water evaporates, voids are formed, weakening the concrete sample's resistance to crack propagation and failure. This conclusion is supported by the findings of Tan et al. and AL-Bawi et al.^88,89^.

##### Assessment of the aging in the samples

**Figure 18:**
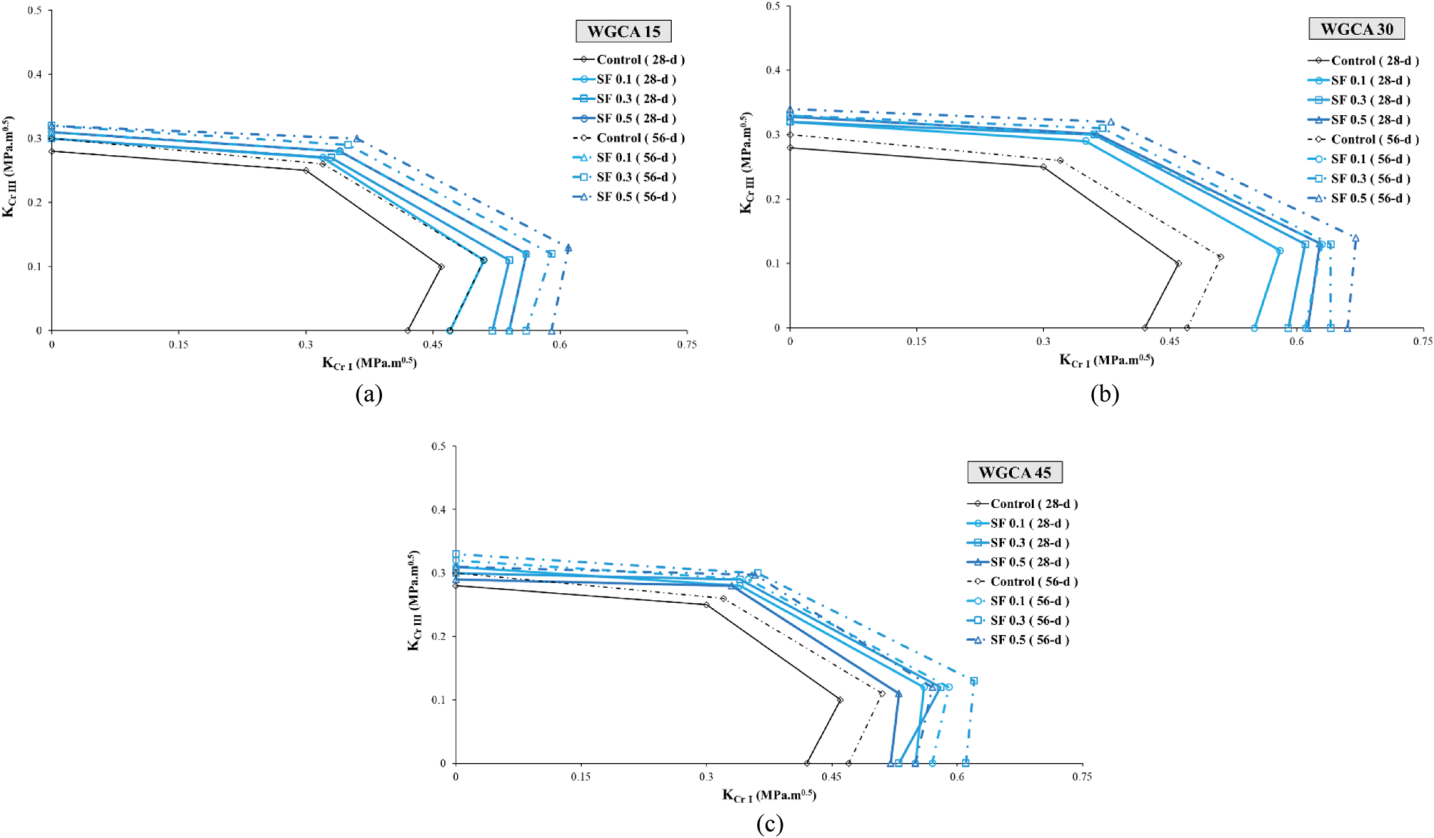
Aging effect of various SF volume fractions on ${\text{K}}_{{{\text{eff}}}}$ of each WGCA volume for (**a**) WGCA 15, (**b**) WGCA 30, and (**c**) WGCA 45.

**Figure 19:**
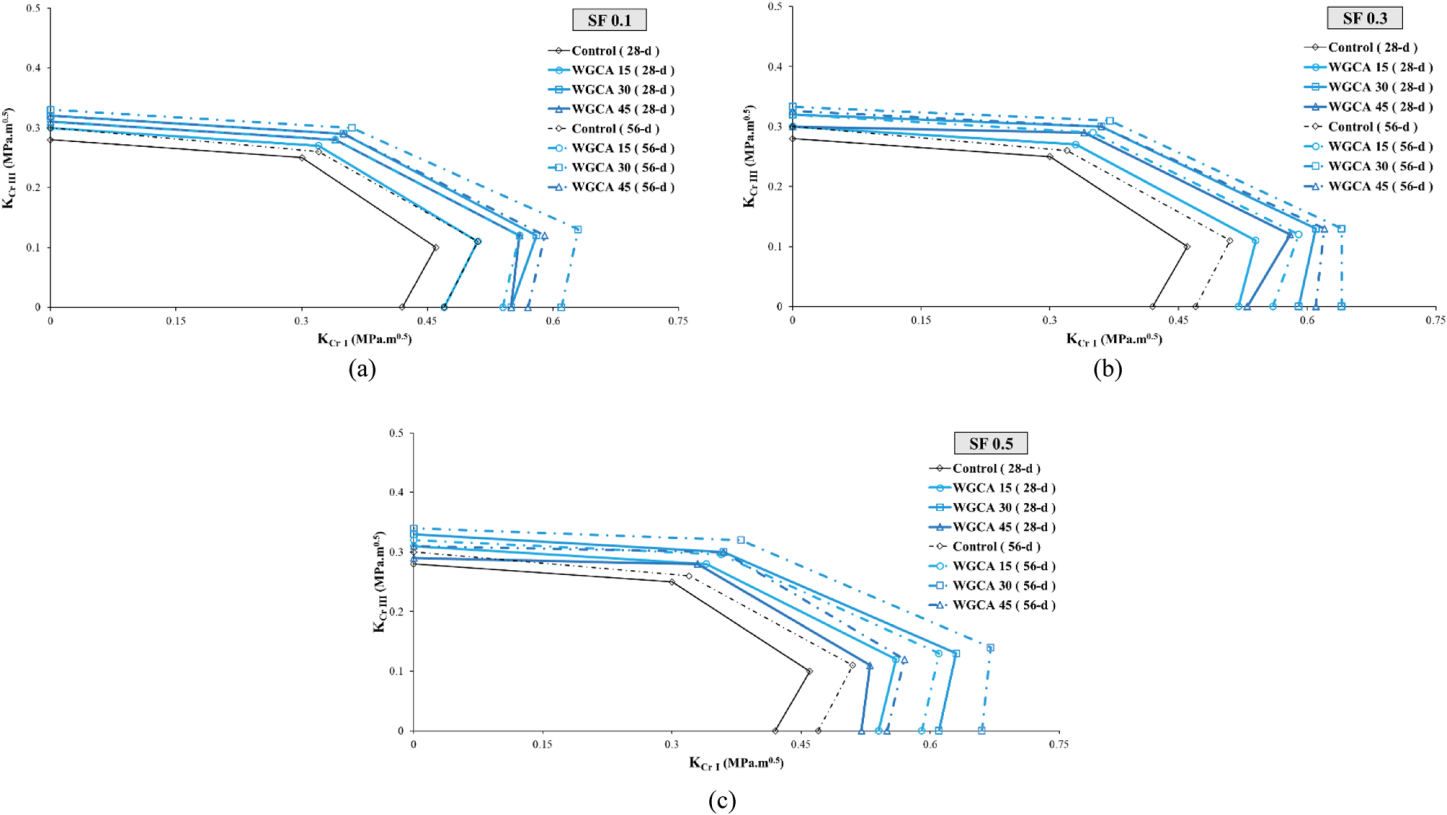
Aging effect of various WGCA volumes on $K_{eff}$ of each SF volume fraction for (**a**) SF 0.1 15, (**b**) SF 0.3, and (**c**) SF 0.5.

Examining the impact of aging on concrete samples holds significant importance, as this factor not only enhances the mechanical properties and durability of concrete but also alters the trajectory of crack propagation. As a result, while in the early ages the crack path traverses the natural aggregate, with the samples' age progression, the path shifts inward within the aggregate. This shift leads to a transformation in the fracture surface of the concrete samples, transitioning from a hardened surface to a more yielding one^90^. This phenomenon also impacts concrete samples containing fibers, leading to a shift from fiber pull-out to fiber rupture^91^. In this investigation, the fracture surfaces of the samples exhibited instances of torn fibers, along with the stretching of fibers that had lost their hooked ends and consequently straightened. According to Beygi et al., this aging phenomenon is attributed to completing of the hydration process and the reinforcement of the interfacial transition zone^90^. The alignment of these findings corresponds with the observations made by the researchers in this study. Among the samples incorporating SF and WGCA, the most notable increase in ${\text{K}}_{{{\text{eff}}}}$ value due to aging was observed in pure modes (I and III), as well as in the combined modes I/III and III/I. Specifically, the WG45SF0.3, WG15SF0.1, and WG45SF0.5 samples exhibited growth rates in their ${\text{K}}_{{{\text{eff}}}}$ values of 15.09%, 10%, 9.62%, and 9.3%, respectively. The impact of increasing the curing period from 28 to 56 days on the fracture toughness of SFR-SCC samples containing WGCA is most pronounced in pure mode I, with an average increase of 9.32%. Similarly, the effect of aging on K_eff_ values in modes I/III, III/I, and III is 7.74%, 5.26%, and 4.73%, respectively. These results indicate that aging has the least impact on pure mode III compared to other modes. Figure 15 clearly demonstrates the significant and increasing effect of extended curing period on the critical load values of the samples. Figures 18 and 19 depict the range of safety of samples prepared under four loading modes, considering the influence of both fibers and glass. The illustrated figures provide a clear insight into the influence of aging on the fracture toughness of the samples. In Fig. 18a,b, the widest range is observed for the SF0.5 (56-d) sample, while in Fig. 18c, it is the SF0.3 (56-d) sample. Additionally, the largest safe zone among all figures in Fig. 19 is attributed to the WGCA 30 (56-d) sample. It is advisable to examine and analyze these diagrams to assess the behavior of materials and structural components prior to commencing the construction process^44^.

##### Evaluating results of loading modes

**Figure 20:**
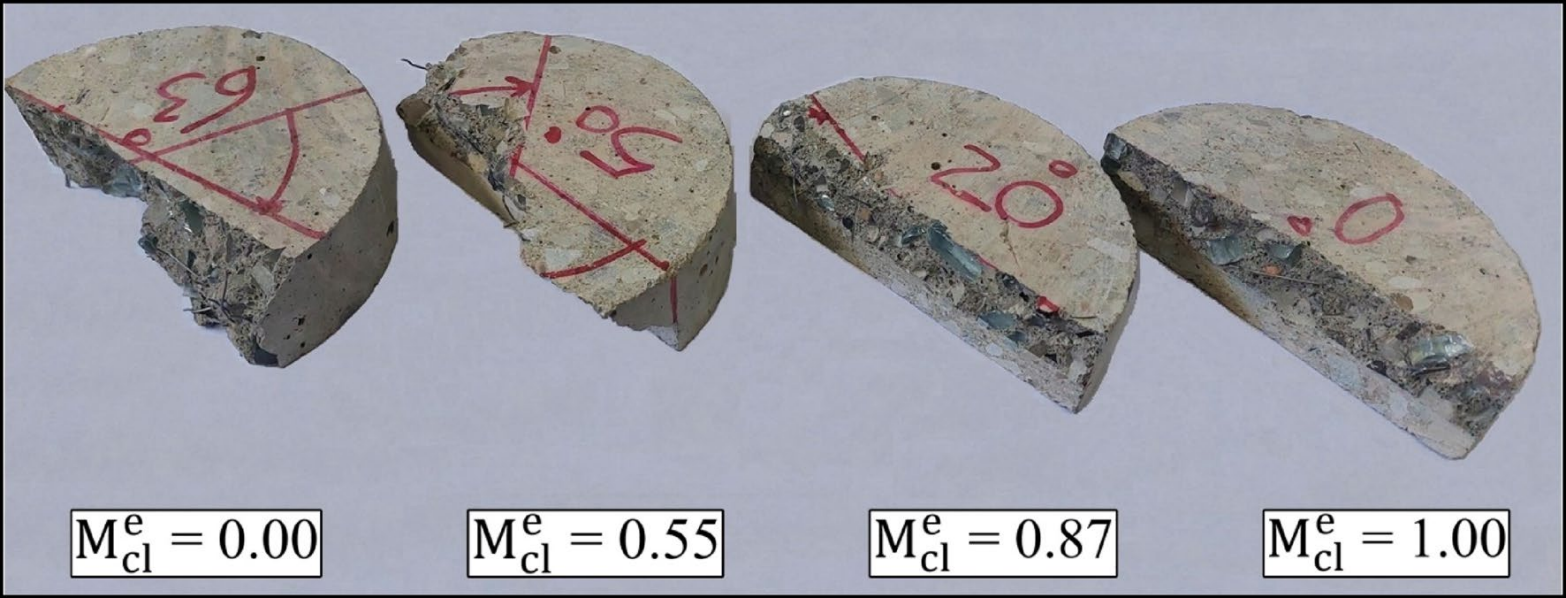
The specimens’ failure path in different loading modes.

**Figure 21:**
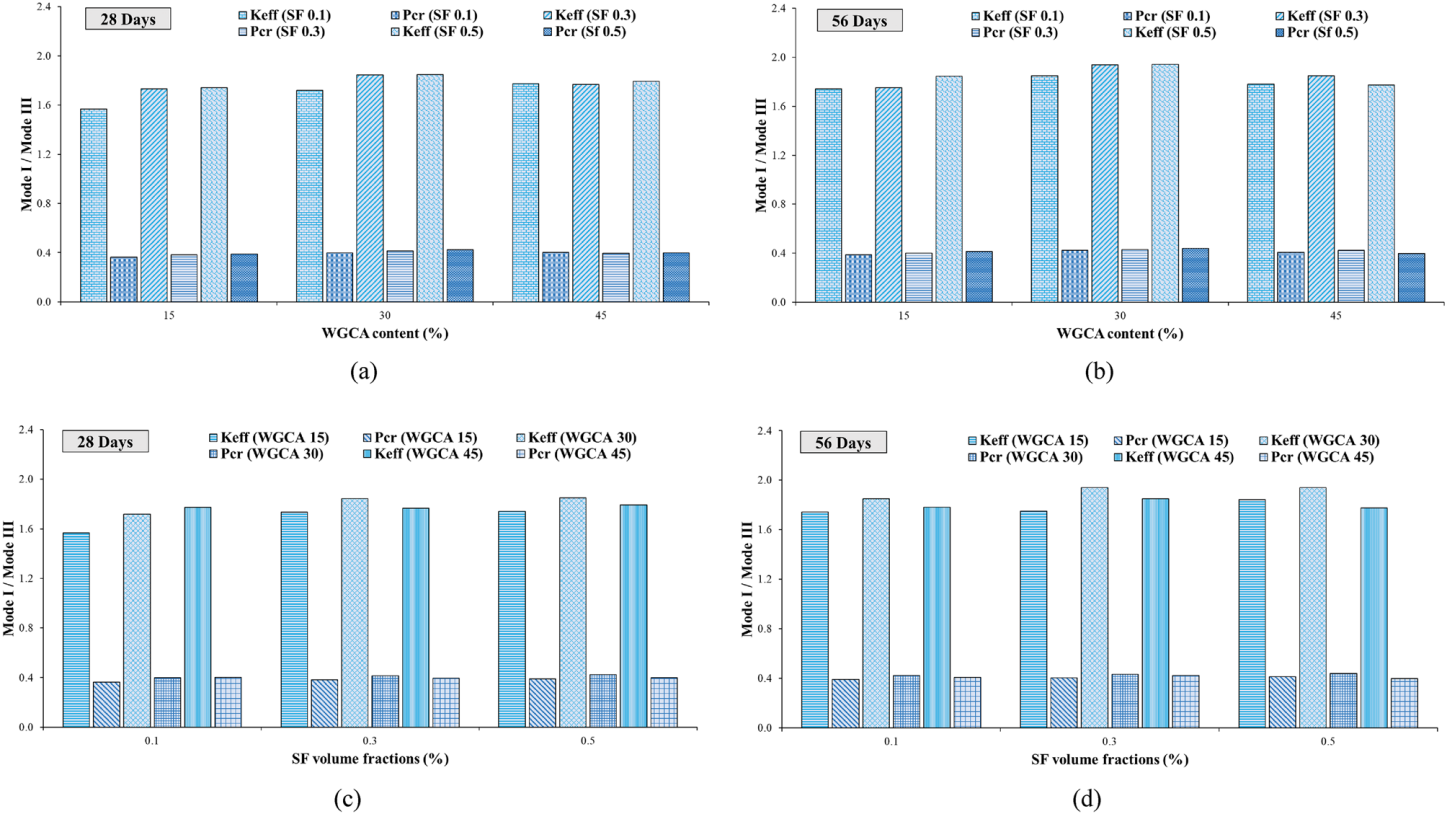
Comparing pure mode I and III fracture parameters of specimens by the separate effect on SF (**a**–**b**) and WGCA (**c**–**d**) volumes at different ages.

Because of the heterogeneous composition of concrete, arising from the amalgamation of diverse components with distinct structures and attributes, coupled with its brittle tendency to fracture under critical loads, this material is particularly susceptible to crack propagation in failure modes (III/II/I)^56^. For a more comprehensive grasp of the failure trajectory in pure modes and their amalgamation, the halves of the 28-day WG45SF0.5 sample are depicted in Fig. 20. Based on the Fig. 20, when the torsional loading (pure mode III) is reduced in favor of tensile loading (pure mode I), the failure path of the samples tends to move towards the primary crack, causing the crack surface to decrease from its maximum value to the minimum value observed in pure mode I. Similar findings have been reported by researchers in previous studies conducted on concrete and asphalt mixtures^44,60,92–94^. The ratio between the effective stress intensity factor (K_eff_) and the critical failure load (P_Cr_) for pure mode I compared to pure mode III, considering the influence of SF and WGCA, is illustrated in Fig. 21. The ratio values for K_eff_ and P_Cr_ of the control sample are 1.5 and 0.34 at 28 days, and 1.56 and 0.35 at 56 days. Figure 21 indicates that the highest ratios of K_eff_ and P_Cr_ among all SFR-SCC samples containing WGCA at both 28 and 56 days belong to the WG30SF0.5 sample, with values of 1.85 and 1.94 for K_eff_, and 0.42 and 0.44 for P_Cr_. On the other hand, the 28 and 56-day samples of WG15SF0.1 exhibit the lowest ratios, with values of 1.57 and 1.74 for K_eff_, and 0.36 and 0.38 for P_Cr_, respectively. Based on Fig. 21, the K_eff_ values for pure mode I in samples containing glass and fibers are 1.57 to 1.94 times higher compared to pure mode III at both ages. However, the P_Cr_ ratio for these samples in pure mode I ranges from approximately 0.36–0.44 compared to pure mode III. This suggests that although the highest critical force value for samples with an initial crack is associated with pure mode III, these samples exhibit weak failure behavior under torsional loading. Therefore, engineers and designers consider pure mode III as a critical loading condition in their analysis and designs, as this parameter directly impacts the lifespan and durability of structures and objects^56^.

**Figure 22:**
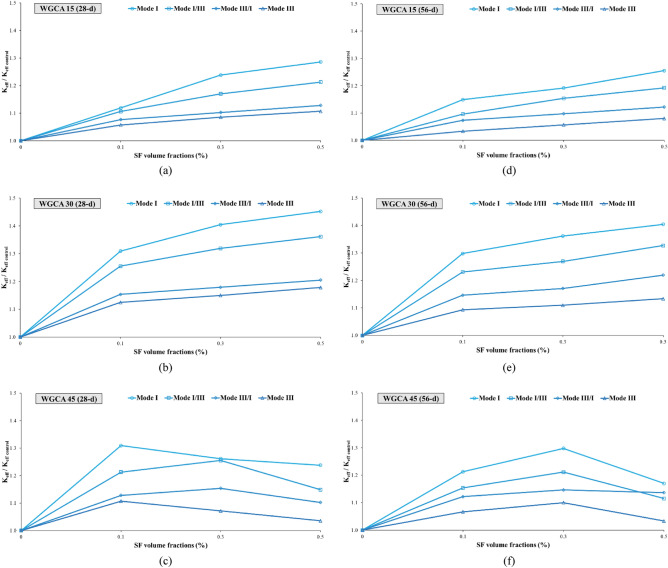
Comparing the effect of different SF volumes on the normalized ${\text{K}}_{{{\text{eff}}}} /{\text{K}}_{{{\text{eff}}_{{\text{ control}}} }}$ ratio of each WGCA volume at different ages.

**Figure 23:**
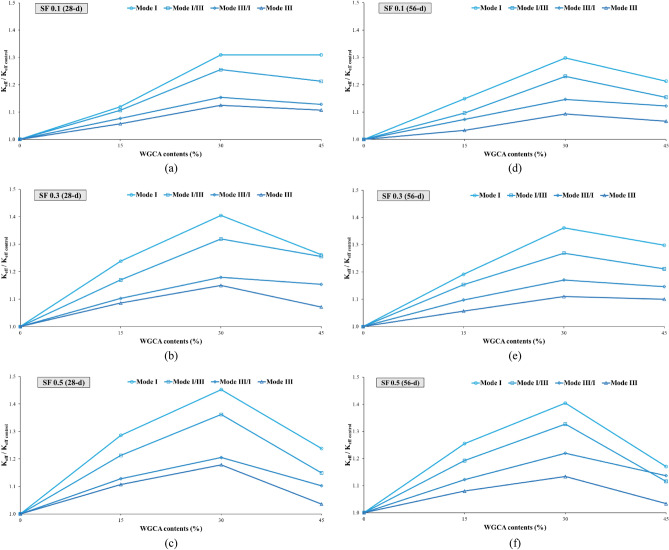
Comparing the effect of different WGCA volumes on the normalized ${\text{K}}_{{{\text{eff}}}} /{\text{K}}_{{{\text{eff}}_{{\text{ control}}} }}$ ratio of each SF volume at different ages.

**Figure 24:**
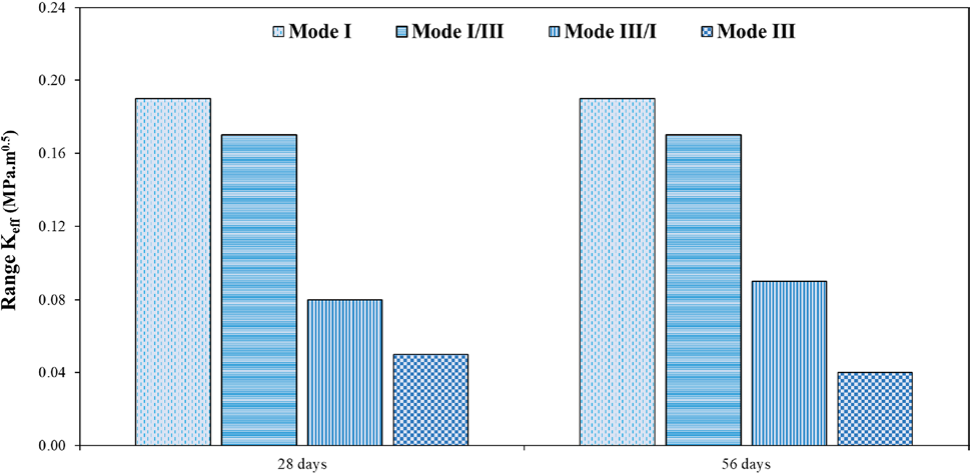
The range of ${\text{K}}_{{{\text{eff}}}}$ changes of SFR-SCC specimens containing WGCA.

To gain a deeper understanding of the variations in the effective stress intensity coefficient of SFR-SCC samples incorporating waste glass in comparison to the control sample, the K_eff_ values of these samples have been normalized with respect to the K_eff_ value of the control sample. The influence of both steel fibers (SF) and waste glass coarse aggregate (WGCA) on these variations is presented separately in Figs. 22 and 23. It is evident that all the ratio values are greater than that of the control sample, with the range of these changes spanning from 3.33 to 45.24%. The highest and lowest rates of increase in changes among all samples are observed in fracture toughness for pure modes I and III. According to Fig. 22, the inclusion of steel fibers (SF) in the samples amplifies the changes in all failure modes, except for the modes of WGCA 45 (28-d) samples containing 0.3% and 0.5% SF, as well as WGCA 45 (56-d) samples containing 0.5% SF, which exhibit a decrease compared to their respective preceding samples. Examining the failure mode I and III of concrete samples containing the effect of synthetic forta-ferro fiber (SFF), Aliha et al. stated that the ratio of changes in K_eff_/K_effcontrol_ to the addition of 0.3% SFF fibers, in both modes Net I and III has had an upward and increasing trend^58^. This factor shows that the presence of fibers in concrete increases the bearing capacity and raises the value of P_Cr_ compared to the reference sample, this finding was also observed in this study. According to Fig. 23, the effect of replacing WGCA up to 30% on 28 and 56 days old concrete samples was positive and increasing in all modes. However, once the substitution level reached 45%, it hindered the growth of changes in all fiber-containing samples. Mousavi et al. observed an increase in the changes of fracture toughness in self-compacting mortar (SCM) samples containing waste glass fine grain (WGFA) compared to the control sample at 28 and 56 days, with replacement values ranging from 20 to 40%^44^. The range of changes, which represents the difference between the minimum and maximum values of the effective stress intensity coefficient for all samples across their respective failure modes, is shown in Fig. 24. The highest difference in this range corresponds to pure mode I, while the lowest difference is associated with pure mode III. As depicted in Fig. 24, the WGCA/SF interaction effect on the fracture toughness is most pronounced in pure mode I compared to the other modes. Notably, the transition from a 28-day processing age to 56 days did not yield a considerable effect on the range of alterations in the effective stress intensity coefficient across the samples. Similar conclusions were reported by various researchers^44,45,90,95^.

**Figure 25:**
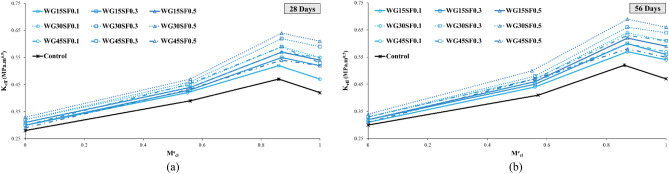
Variations of Kef versus Me for SFR-SCC specimens containing WGCA at 28 (**a**) and 56 (**b**) ages.

Figure 25 illustrates the alterations in the effective stress intensity coefficient for all samples in relation to their share contributions of different loading modes (${\text{M}}_{{{\text{cl}}}}^{{\text{e}}}$) at both 28 and 56-day ages. As depicted by Fig. 25, the most significant increase and decrease in ${\text{K}}_{{{\text{eff}}}}$ values across different modes, compared to pure mode I, were observed in mode I/III and pure mode III, respectively. The relatively limited effectiveness in impeding crack growth and propagation in pure mode III (tearing or anti-plane shear) has led researchers to deem situations where this mode is dominant as particularly critical^83^. The reduction in ${\text{K}}_{{{\text{eff}}}}$ from pure mode III to pure mode I was 33.33% and 36.17% for the 28 and 56-day control samples, respectively. In contrast, this reduction for all 28 and 56-day SFR-SCC samples containing WGCA averaged around 42.85% and 45.27%, respectively.

## Conclusion

The objective of this study is to address environmental concerns associated with the extraction of natural aggregates from mines and the disposal of glass waste in the environment. To accomplish this goal, a scientifically and dependable approach was employed to develop an eco-friendly steel fiber reinforced self-compacting concrete (Eco-SFR-SCC). The concrete was produced by incorporating waste tempered glass coarse aggregates at various percentages (15%, 30%, and 45%). The investigations were carried out at two curing durations of 28 and 56 days. Furthermore, it is important to note that this study focused on initial pre-cracks and did not consider the fiber-bridge effect at the initial crack level. In this context, the research examined the flowability and workability of concrete in its fresh state, as well as its compressive strength and critical stress intensity coefficient in pure mode I, mixed mode I/III, mixed mode III/I, and pure mode III. The summarized findings are presented below:

- The combined effect of SF and WGCA on the properties of fresh concrete in all samples exhibits a negative and decreasing trend when compared to the control sample. The sample with the most significant reduction in slump flow diameter and blockage ratio is WG45SF0.5, showing decreases of 18.46% and 16.49% respectively. Notably, there is a strong correlation between the parameters of fresh concrete, as evidenced by an R^2^ value of 0.89.
- The compressive strength of all SFR-SCC samples incorporating WGCA at both curing durations surpasses that of the control sample. The highest percentage increase in compressive strength at 28 and 56 days is observed in the sample where 15% WGCA is replaced, and 0.5% SF is added (WG15SF0.5). The increases are recorded as 17.57% and 23.01% respectively. Conversely, in samples containing 45% WGCA, there is a declining trend in compressive strength with the increase in volume fraction of SF. The lowest increments at both curing durations are observed in the WG45SF0.5 sample, with percentages of 2.13% (28 days) and 1.78% (56 days).
- The P_Cr_ and K_eff_ values of all samples containing both SF and WGCA at both curing durations are higher compared to the control sample. Among all the samples, the pure mode III and combined mode I/III exhibit the highest values for P_Cr_ and K_eff_, while the pure modes I and III demonstrate the lowest values for these parameters.
- Increasing the SF volume (ranging from 0.1 to 0.5%) up to 30% substitution of WGCA leads to augmented ${\text{P}}_{{{\text{Cr}}}}$ and ${\text{K}}_{{{\text{eff}}}}$ across all age ranges. Nevertheless, this increment, when applied to samples containing 45% WGCA, results in a decline in both ${\text{P}}_{{{\text{Cr}}}}$ and ${\text{K}}_{{{\text{eff}}}}$ in comparison to previous replacements. Among the various combinations, the most pronounced and minimal impacts of SF and WGCA on critical load and fracture toughness values are evident in the WG30SF0.5 and WG15SF0.1 samples, respectively.
- Considering the significance of the fracture behavior observed in pure mode III under torsional loading, the influence of SF and WGCA on the fracture toughness of SCC at 28 and 56 days is noteworthy. In comparison to the control sample, the incorporation of SF and WGCA in this concrete yield an average increase of 10.31% and 7.77% in fracture toughness, respectively, across both age intervals.
- The aging process and the mode of loading across different scenarios not only effect the loadbearing capacity and safety zone of the samples but also impact the trajectory of their growth, propagation, and extent of cracking. Among these influences, the most marginal rise in the effect of aging on various modes of fracture toughness was observed in pure mode III, with an increment of 4.73%.

According to the experimental work done in this study, it was found that the simultaneous effect of WGCA and SF on self-compacting concrete improves and increases the performance of hardened properties of this concrete, compressive strength and fracture parameters, compared to the control sample. SFR-SCC containing WGCA can be used in concrete structures that, in addition to increasing the load-bearing capacity against dynamic loads that cause vibration in the structure, such as; Earthquake load, structural element weight reduction and project costs are considered, should be used.

## Data Availability

The datasets used and/or analyzed during the current study available from the corresponding author on reasonable request.
